# Comparative Genomics of Antibiotic-Resistant Uropathogens Implicates Three Routes for Recurrence of Urinary Tract Infections

**DOI:** 10.1128/mBio.01977-19

**Published:** 2019-08-27

**Authors:** Robert Thänert, Kimberly A. Reske, Tiffany Hink, Meghan A. Wallace, Bin Wang, Drew J. Schwartz, Sondra Seiler, Candice Cass, Carey-Ann D. Burnham, Erik R. Dubberke, Jennie H. Kwon, Gautam Dantas

**Affiliations:** a The Edison Family Center for Genome Sciences and Systems Biology, Washington University School of Medicine, St. Louis, Missouri, USA; b Department of Pathology and Immunology, Washington University School of Medicine, St. Louis, Missouri, USA; c Division of Infectious Diseases, Washington University School of Medicine, St. Louis, Missouri, USA; d Department of Pediatrics, Washington University School of Medicine, St. Louis, Missouri, USA; e Department of Molecular Microbiology, Washington University School of Medicine, St. Louis, Missouri, USA; f Department of Biomedical Engineering, Washington University, St. Louis, Missouri, USA; Department of Veterinary Medicine; Massachusetts Institute of Technology; Weill Cornell Medical College

**Keywords:** antibiotic resistance, clonal tracking, comparative genomics, recurrence, urinary tract infection

## Abstract

The rise of antimicrobial resistance in uropathogens has complicated the management of urinary tract infections (UTIs), particularly in patients who are afflicted by recurrent episodes of UTIs. Antimicrobial-resistant (AR) uropathogens persistently colonizing individuals at asymptomatic time points have been implicated in the pathophysiology of UTIs. The dynamics of uropathogen persistence following the resolution of symptomatic disease are, however, mostly unclear. To further our understanding, we determined longitudinal AR uropathogen carriage and clonal persistence of uropathogenic Escherichia coli, Proteus mirabilis, and Klebsiella pneumoniae isolates in the intestinal and urinary tracts of patients affected by recurrent and nonrecurrent UTIs. Clonal tracking of isolates in consecutively collected urine and fecal specimens indicated repeated transmission of uropathogens between the urinary tract and their intestinal reservoir. Our results further implicate three independent routes of recurrence of UTIs: (i) following an intestinal bloom of uropathogenic bacteria and subsequent bladder colonization, (ii) reinfection of the urinary tract from an external source, and (iii) bacterial persistence within the urinary tract. Taken together, our observation of clonal persistence following UTIs and uropathogen transmission between the intestinal and urinary tracts warrants further investigations into the connection between the intestinal microbiome and recurrent UTIs.

## Abstract (precis)

The increasing antimicrobial resistance of uropathogens is challenging the continued efficacy of empiric antibiotic therapy for UTIs, which are among the most frequent bacterial infections worldwide. It has been suggested that drug-resistant uropathogens could persist in the intestine after the resolution of UTI and cause recurrences following periurethral contamination. A better understanding of the transmission dynamics between the intestinal and urinary tracts, combined with phenotypic characterization of the uropathogen populations in both habitats, could inform prudent therapies designed to overcome the rising resistance of uropathogens. Here, we integrate genomic surveillance with clinical microbiology to show that drug-resistant clones persist within and are readily transmitted between the intestinal and urinary tracts of patients affected by recurrent and nonrecurrent UTIs. Thus, our results advocate for understanding persistent intestinal uropathogen colonization as part of the pathophysiology of UTIs, particularly in patients affected by recurrent episodes of symptomatic disease.

## INTRODUCTION

Urinary tract infections (UTIs) are among the most common bacterial infections, occurring more frequently in women, half of whom will experience at least one episode in their lifetime (1). UTIs result in estimated yearly costs of $3.5 billion and 10.5 million office visits in the United States alone (2). Besides gender, a history of UTIs, sexual activity, diabetes, obesity, and genetically determined susceptibility are major risks factors associated with UTIs (2, 3). A UTI is diagnosed when urinary symptoms coincide with evidence of urinary tract inflammation and urine cultures are positive for a known uropathogen above a defined threshold (commonly 10^5^ CFU/ml) (4, 5). The majority of UTIs are caused by uropathogenic Escherichia coli (UPEC) strains, which are specifically adapted to colonize the urinary tract (6). Besides UPEC, strains of Klebsiella pneumoniae, Staphylococcus saprophyticus, Enterococcus faecalis, and Proteus mirabilis are frequent causes of UTIs (2, 7).

Approximately 25% of women suffering from UTIs experience at least one recurrence of symptomatic UTI (rUTI) within a window of 6 months following the initial infection (8). The model for the majority of recurrences postulates that uropathogens persist in an intestinal reservoir and recolonize the urinary tract following periurethral contamination (1, 9). Supporting this hypothesis, previous studies have shown that the strain of UPEC associated with symptomatic UTIs is commonly found in fecal specimens collected from the same patient (10, 11). Besides intestinal persistence, quiescent intracellular reservoirs (QIRs) established by UPEC strains in the bladder epithelium, reinfection from external sources, and asymptomatic bacteriuria have been implicated in the pathophysiology of rUTIs (12–16). The dynamics of bacterial persistence during the succession of resolution of the UTI, asymptomatic latency, and the recurrence of symptomatic infection, knowledge of which could be used to help assess the relative importance of different routes of rUTIs and guide the development of novel interventions, have not yet been established.

With appropriate treatment, uncomplicated UTIs are commonly considered a benign condition with no long-term medical consequences (17). However, these infections can cause pain, reduce the life quality of afflicted individuals (18), and progress to pyelonephritis or sepsis if the uropathogen ascends to the kidneys or enters the bloodstream (2). Thus, antimicrobial therapy remains a mainstay of treatment.

The rise of antimicrobial resistance in major uropathogens has complicated the standard treatment of UTIs (6, 19). Today, antimicrobial-resistant (AR) strains of bacteria associated with UTIs are frequently resistant to the most commonly used drugs (6), and plasmids carrying the genes for extended-spectrum β-lactamases (ESBLs) have rapidly spread within the *Enterobacteriaceae* family (2). Alarmingly, ESBL-producing UPEC strains are now also occurring in patients with no discernible risk factors for the carriage of antimicrobial-resistant organisms (AROs), such as exposure to health care facilities (20). The continued efficacy of empirical antibiotic therapy is particularly complicated in patients afflicted by chronic rUTIs, who frequently have a history of repeated antibiotic exposure that can lead to increasing antimicrobial resistance in uropathogens. For these patients, strategies that can guide prudent empirical treatment are urgently needed. The prevailing clinical concept is to treat individuals with a history of rUTIs based on the antibiotic susceptibility profiles of uropathogens isolated during prior episodes. This concept, however, requires assessment of the uropathogen populations colonizing patients for existing genomic heterogeneity, as this can impact population-level functional profiles (21, 22).

Genetic variation within pathogen populations can result from cocolonizing bacterial lineages or the genetic diversification of pathogens under the selective pressures encountered within their environment (22). Importantly, intrapatient strain diversity can increase the standing diversity of clinically relevant features like virulence (21, 23) or antibiotic resistance (24, 25), potentially hampering treatments that are based on the patient’s medical history. To date, there is limited knowledge about the extant genetic diversity within the antibiotic-resistant uropathogen populations colonizing individual patients and clonal persistence following the conclusion of antimicrobial therapy.

In this study, we define the genetic relatedness of 109 AR isolates of the major uropathogens E. coli, P. mirabilis, and K. pneumoniae isolated from urine and fecal specimens that were collected longitudinally from 14 patients affected by rUTIs and non-rUTIs at symptomatic and asymptomatic time points. We combined clonal tracking with semiquantitative culturing of urine and stool specimens to provide a temporally resolved characterization of the dynamics of bacterial clearance and persistence following UTIs. Our data highlight substantial interpatient differences in the pathophysiology of rUTIs caused by AR uropathogens and confirm reinfection from external sources, urinary persistence, or relapse from the intestinal reservoir to be three different routes for rUTIs.

## RESULTS

### Study cohort and microbiology.

Fourteen patients (median age, 63 years; age range, 37 to 88 years) with symptomatic UTIs caused by AR uropathogens were enrolled in this study (Table 1). Of these, seven patients (50%) experienced up to two episodes of rUTIs during a follow-up period of 180 days after the conclusion of antimicrobial treatment (Table 1). Three different uropathogens were cultured from urine samples obtained at the time of the UTI; E. coli was isolated from the urine of 12 patients (5 patients with non-rUTIs, 7 patients with rUTIs), Klebsiella pneumoniae was identified in one case (one non-rUTI), and Proteus mirabilis was cultured from samples from two patients (one with a non-rUTI, one with a rUTI; Table 1). Both P. mirabilis and E. coli were cultured from diagnostic urine samples by the research lab for patient 10, but only E. coli was recovered from the same urine specimen by the clinical microbiology lab.

**TABLE 1:** Cohort summary^*a*^

Patient identifier^*b*^	Age (yr)	Antibiotic treatment	Episode no.	MLST ST	Uropathogen	Resistance												
						GEN	MIN	NIT	SAM	FEP	CAZ	ATM	CRO	CFZ	AMP	CIP	SXT	DOX
1	37	Ertapenem	1	405	E. coli	R	S	S	R	R	R	R	R	R	R	R	R	I
2	66	Ciprofloxacin	1	12	E. coli	S	S	S	R	S	R	S	R	R	R	S	S	R
3	42	Nitrofurantoin	1	551	K. pneumoniae	S	S	S	R	S	I	I	R	R	R	R	R	R
4	62	Nitrofurantoin	1	131	E. coli	S	S	S	S	—	S	S	R	R	R	R	S	R
			2	—	E. coli	—	—	—	—	—	—	—	—	—	—	—	—	—
5	54	Nitrofurantoin	1	131	E. coli	S	S	S	S	R	R	R	R	R	R	R	S	I
			2	69	E. coli	S	—	S	—	S	S	—	S	S	R	S	S	—
6	43	Nitrofurantoin	1	131	E. coli	R	S	S	R	R	I	I	R	R	R	R	S	I
			2	—	E. coli	—	—	—	—	—	—	—	—	—	—	—	—	—
8	72	Meropenem	1	131	E. coli	S	S	S	R	R	R	R	R	R	R	R	R	R
9	88	Doxycycline	1	131	E. coli	S	S	S	I	R	R	R	R	R	R	R	R	S
10^*c*^	66	Ertapenem	1	131	E. coli	S	S	S	R	R	R	R	R	R	R	R	R	R
			1		P. mirabilis	R	R	R	R	I	S	S	R	R	R	R	R	R
13	62	Nitrofurantoin	1	131	E. coli	S	S	S	R	R	R	R	R	R	R	R	R	R
			2	131	E. coli	S	S	S	R	R	R	R	R	R	R	R	R	R
			3	131	E. coli	S	S	S	R	R	R	R	R	R	R	R	R	R
14	67	Meropenem	1		P. mirabilis	S	R	R	S	I	S	S	R	R	R	R	R	R
			2		P. mirabilis	S	R	R	R	I	S	S	R	R	R	R	R	R
15	64	Ciprofloxacin, co-trimoxazole	1	131	E. coli	S	S	S	S	S	S	S	S	I	R	R	S	S
16	74	Ciprofloxacin, nitrofurantoin	1	131	E. coli	S	S	S	I	R	R	R	R	R	R	R	R	S
			2	—	E. coli	—	—	—	—	—	—	—	—	—	—	—	—	—
18	43	Cefalexin	1	131	E. coli	R	S	S	S	S	S	S	S	I	R	R	R	R
			2	131	E. coli	R	S	S	I	S	S	S	S	R	R	R	R	R

a Abbreviations and symbols: GEN, gentamicin; MIN, minocycline; NIT, nitrofurantoin; SAM, ampicillin-sulbactam; FEP, cefepime; CAZ, ceftazidime; ATM, aztreonam; CRO, ceftriaxone; CFZ, cefazolin; AMP, ampicillin; CIP, ciprofloxacin; SXT, trimethoprim-sulfamethoxazole; DOX, doxycycline; R, resistant, S, susceptible; I, intermediate; —, missing data for isolates unavailable for analysis.

b All patients were female.

c Patient 10 received a kidney transplant.

**FIG 1:**
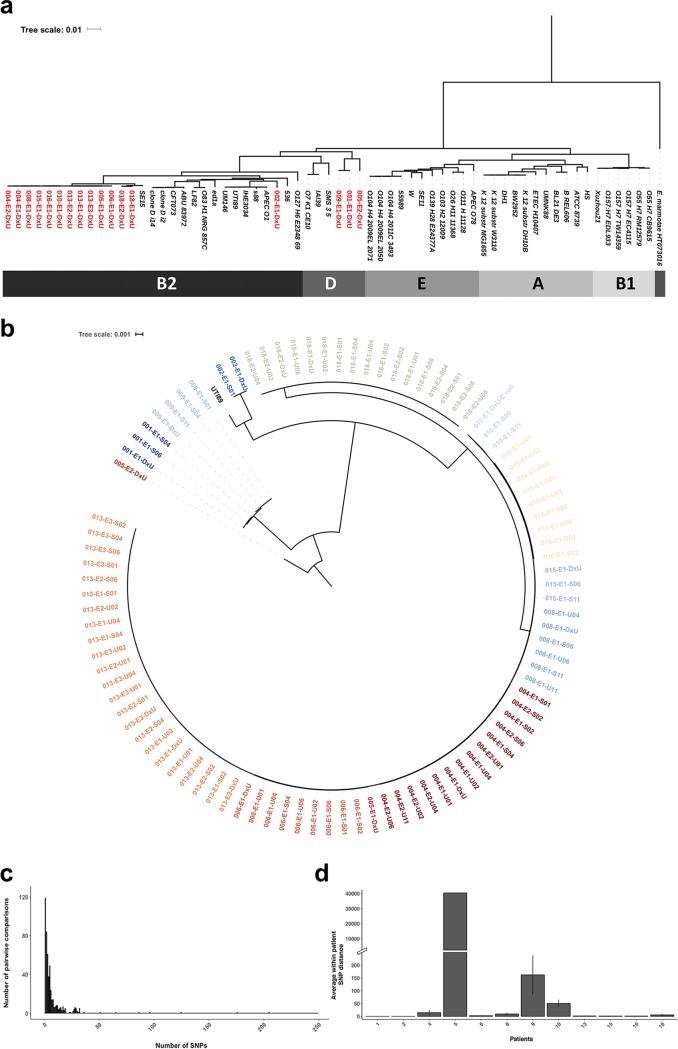
The clonal diversity of the uropathogen population varies between individual patients. (a) UTI-associated E. coli strains belong to the major phylogroups D and B2. The maximum likelihood phylogenetic tree includes all available E. coli isolates cultured from diagnostic urine samples and publicly available reference strains. Diagnostic isolates are depicted in red; publicly available reference genomes are indicated in black. Escherichia marmotae strain HT073016 was added as an outgroup (italics). (b) Phylogenetic reconstruction indicates that the E. coli isolates clustered by patient. The maximum likelihood phylogenetic tree includes all available E. coli isolates cultured from urine and stool samples. Isolates are colored according to patient. E. coli strain UTI89 was added as a reference. (c) Isolates from the same patient generally differed by <50 SNPs. The histogram shows the number of SNPs identified for all within-patient pairwise comparisons of E. coli isolates. (d) The average number of SNPs identified in all pairwise comparisons of E. coli isolates collected from each patient. Error bars depict standard deviations.

Consistent with previous reports (2), E. coli was the dominant uropathogen associated with UTIs in our study (12/14 patients). *In silico* multilocus sequence typing (MLST) and core genome alignment of all E. coli strains cultured from diagnostic urine specimens (*n *=* *17) and a set of reference strains (*n *=* *46; see Text S1 in the supplemental material) showed that the isolated ESBL-producing E. coli strains belonged to the B2 (82.4%, 14 of 17) or D (17.8%, 3 of 17) phylogroup (Fig. 1a) and were predominantly sequence type 131 (ST131) (13/17 isolates) (Table 1). Maximum likelihood trees (based on 2,074 core genes that shared at least 95% nucleotide identity) supported the MLST/phylogroup data, as all isolates identified as phylogroup B2 or D clustered with reference strains from their respective phylogroup (Fig. 1a). Additionally, we found that the majority of AR E. coli isolates collected at the time of diagnosis (DxU) of a recurrence (3/4) were closely related to the clone isolated during the initial episode of UTI (same-strain rUTIs). In a single case (patient 5), the isolate associated with the recurrence fell into a phylogroup (phylogroup D) different from that of the strain associated with the initial episode of UTI (phylogroup B2).

10.1128/mBio.01977-19.9 TEXT S1 Detailed description of the methods used during WGS *in silico* analysis. Download Text S1, DOCX file, 0.02 MB. Copyright © 2019 Thänert et al. 2019 Thänert et al. This content is distributed under the terms of the Creative Commons Attribution 4.0 International license.

### WGS reveals that most patients are stably colonized by clonal uropathogen populations.

We performed whole-genome sequencing (WGS) on 95 E. coli isolates, 13 P. mirabilis isolates, and 1 K. pneumoniae isolate cultured from urine and stool specimens at symptomatic and asymptomatic time points (Table S1). To characterize the genetic relatedness of the dominant uropathogen, E. coli, we constructed a species-specific core genome comprising the genes shared at ≥95% identity. Phylogenetic reconstruction using all E. coli isolates indicated that the isolates clustered by patient rather than by habitat (Fig. 1b). To assess the genetic diversity of the within-patient AR uropathogen populations colonizing individual patients, we identified single nucleotide polymorphism (SNP) distances by alignment of raw reads to the reference assembly of an isolate collected from the same patient (see Materials and Methods). Generally, SNP distances between isolates collected from the same patient were small (Fig. 1c), with the E. coli populations of 6/12 patients (patients 1, 2, 6, 13, 15, and 16) having an average of less than 5 SNPs (Fig. 1d), indicating that individual patients are frequently colonized by a clonal population of AR uropathogens. Isolates clonal with the DxU isolate (SNP distance = 0) were recovered from stool samples from patients 1, 2, and 16 at asymptomatic time points, highlighting that AR uropathogens can stably colonize patients. Moreover, the recovery of clonal isolates from urine and stool samples at asymptomatic time points from multiple patients (patients 6, 8, and 18; Fig. S1) highlights the interconnectedness of the uropathogen populations colonizing the urinary and intestinal tracts. In three of the four patients (patient 13, 14, and 18) with available diagnostic isolates from a recurrence of symptomatic UTI, we found that each episode was associated with the same lineage (<10 SNPs).

10.1128/mBio.01977-19.1 FIG S1 Per patient phylogenetic reconstruction of E. coli lineages. Single nucleotide polymorphism (SNP)-based phylogeny of E. coli isolates collected from the same patients. Neighbor-joining trees were recreated from all variable positions. Yellow squares indicate isolates collected from urine samples; brown squares indicate isolates taken from stool samples. Download FIG S1, TIF file, 0.5 MB. Copyright © 2019 Thänert et al. 2019 Thänert et al. This content is distributed under the terms of the Creative Commons Attribution 4.0 International license.

10.1128/mBio.01977-19.8 TABLE S1 Patient samples, culture results, urine 16S rRNA gene data identifiers, and isolate identifiers. Download Table S1, DOCX file, 0.05 MB. Copyright © 2019 Thänert et al. 2019 Thänert et al. This content is distributed under the terms of the Creative Commons Attribution 4.0 International license.

Notably, E. coli isolates collected from three patients (patient 5, 9, and 10) exhibited substantially more mutations (>50 SNPs; Fig. 1d). While the isolation of clones from two different phylogroups during subsequent episodes explained the high SNP distance for patient 5, the E. coli populations of patients 9 and 10 showed a substantially elevated number of mutations between isolates belonging to the same phylogroup (average SNP distances, 162.16 and 50.66, respectively). Analysis of the SNP identity for the E. coli populations of patients 9 and 10 showed no substantial change in the transition/transversion rate or the nature of the base substitutions (Fig. S2), both of which are frequently associated with hypermutating lineages, compared to the E. coli communities collected from all other patients.

10.1128/mBio.01977-19.2 FIG S2 Base substitution frequencies observed in the genomes of the E. coli isolates sequenced in this study. (a) Frequency of single nucleotide polymorphisms classified by the specific nature of the base substitution. Data for isolates collected from patients 9 (dark blue) and 10 (light blue) and the average value for all E. coli isolates from all other patients (black) are depicted. Error bars indicate standard deviations across E. coli populations collected from all other patients. (b) Relative frequency of base transitions and transversion. “Others” indicates the average frequency across E. coli populations from all patients that are not individually depicted. Download FIG S2, TIF file, 0.5 MB. Copyright © 2019 Thänert et al. 2019 Thänert et al. This content is distributed under the terms of the Creative Commons Attribution 4.0 International license.

### Distinct patterns of uropathogen persistence are associated with rUTI.

**FIG 2:**
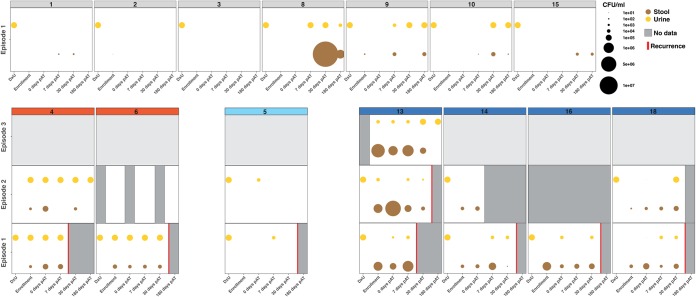
Recurrent urinary tract infections are associated with differences in stool and urine uropathogen density. (Top) Non-rUTI patients; (bottom) patients with rUTIs. Subsequent episodes for rUTI patients are depicted from bottom to top in the bottom panel. Different colonization patterns are indicated by header background colors: red, persistent urinary tract colonization; turquoise, nonpersistent colonization; blue, intestinal blooms. Patient identifiers are given on the top of each panel. The bubble size corresponds to the number of CFU that grew on species-specific selective agar plates (see Materials and Methods). Yellow bubbles correspond to the culture results for urine samples; brown bubbles correspond to the culture results for stool samples. Missing culture data are indicated by gray fields. A red line indicates the time frame in which an rUTI patient experienced a recurrence.

To determine the temporal dynamics of pathogen colonization, we performed semiquantitative culture of stool and urine specimens. The results indicated a trend toward lower AR uropathogen burdens at the asymptomatic time point for patients that did not experience a rUTI during the duration of study enrollment (Fig. 2). Thus, all cultures of post-antimicrobial treatment (pAT) urine and stool specimens were negative for AR uropathogens for 4/7 (57.1%) and 2/7 (28.6%) of all non-rUTI patients, respectively. However, we cultured isolates from the same lineage as the isolate cultured from the diagnostic urine specimen for the majority of non-rUTI patients (71.43%, 5 of 7; Fig. 1d and Fig. S1) at asymptomatic time points, indicating that AR uropathogen colonization alone does not explain all rUTIs.

Integrating information on isolate clonality with the results of semiquantitative culturing revealed three distinct temporal patterns of bacterial clearance and persistence associated with recurrences. First, in patients 4 and 6 (both of whom were colonized with E. coli), recurrence was associated with high urine uropathogen titers that persisted following the conclusion of antimicrobial therapy (Fig. 2). Interestingly, both patients were persistently colonized by isolates from a single lineage, indicating that the causative agent of the urinary tract infection resisted clearance by antibiotic therapy, persisted in the urinary tract, and later caused a recurrence of symptomatic disease.

Second, we observed that recurrences were preceded by several negative pAT urine and stool cultures in patient 5 (who was colonized with E. coli). As the isolate associated with the recurrence fell into a different phylogroup, reinfection from an external source may have been the cause for the rUTI in this patient.

Finally, four cases of rUTI associated with either E. coli (patients 13, 16, and 18) or P. mirabilis (patient 14) showed complex patterns of interspersed negative and positive urine specimens following the conclusion of antimicrobial therapy (Fig. 2). In all four patients, we observed that increased AR uropathogen abundance in fecal specimens preceded positive urine cultures associated with isolates from the same lineage (Fig. 2; Fig. S1 and S3). Moreover, we frequently observed a bloom, defined as a substantially increased ARO abundance relative to that in the previously collected fecal specimen, associated with the fecal specimen collected at a time point preceding or coinciding with the time of detection of the same lineage in urine (e.g., patient 13, episode 2, 0 days pAT; patient 14, episode 1, 7 days pAT; patient 18, episode 1, 0 days pAT). Importantly, episodes of rUTI following such a bloom were caused by the lineage of AR E. coli that we previously isolated during the intestinal bloom in three patients (patients 13, 14, and 18; Fig. 2; Fig. S1 and S3). However, not every bloom was immediately followed by an episode of rUTI (patient 8; Fig. 2) or persistent colonization of the urinary tract (e.g., patient 15, 0 to 7 days pAT), implicating additional factors in the onset of rUTIs.

10.1128/mBio.01977-19.3 FIG S3 P. mirabilis isolates recovered from the same patient belong to a single lineage. (a) Histogram of the number of SNPs identified for all within-patient pairwise comparisons of P. mirabilis isolates. (b) Average number of SNPs identified in all pairwise comparisons of P. mirabilis isolates collected from a given patient. Error bars depict standard deviations. (c) Single nucleotide polymorphism (SNP)-based phylogeny of P. mirabilis isolates collected from the same patients. Neighbor-joining trees were recreated from all variable positions. Yellow squares indicate isolates collected from urine samples; brown squares indicate isolates taken from stool samples. Download FIG S3, TIF file, 0.5 MB. Copyright © 2019 Thänert et al. 2019 Thänert et al. This content is distributed under the terms of the Creative Commons Attribution 4.0 International license.

16S rRNA gene sequencing of the urinary microbiota supported the patterns of bacterial persistence in urine following the conclusion of antimicrobial therapy identified using semiquantitative culturing (Fig. S4a; Data Set S1). We observed consistently high relative abundances of *Enterobacteriaceae* for patients 4 and 6, while increased relative abundances in urine (e.g., patient 10 at 30 days pAT, patient 13 at 30 days pAT) coincided with the increased abundances measured by semiquantitative culturing. Overall, 16S rRNA sequencing results showed a high concordance with culture-based identification of AR uropathogens (Fig. S4d), highlighting that the pathogenic agent was correctly identified by diagnostic urine culturing. We did not identify the specific microbiota compositions at asymptomatic time points with an insignificant uropathogen load (see Materials and Methods) that differentiated patients with rUTIs and those with non-rUTIs (Fig. S4b). Similarly, we did not observe a significant difference between the urinary microbiota composition at matched time points preceding a recurrence (Fig. S4c). This suggests that no specific microbiota signature of asymptomatic urine was associated with recurrence or protection against recurrence in the analyzed set of patients.

10.1128/mBio.01977-19.4 FIG S4 16S rRNA sequencing of longitudinally collected urine samples. (a) Stacked bar graph of family-level relative abundances in longitudinally collected urine samples. Patient identifiers are given as colored annotations on top of the bar graph. Red lines indicate the time point of recurrence in rUTI patients. (b) Principal-coordinate analysis of the taxonomic bacterial composition in urine samples collected at asymptomatic time points that showed insignificant pathogen colonization. Filled circles indicate samples from rUTI patients; open circles highlight samples collected from patients with non-rUTIs. (c) Stacked bar graph of family-level relative abundances in time-matched urine samples from patients with rUTIs and non-rUTIs. Patient groups (non-rUTI, rUTI) are given as colored annotations on top of the bar graph. Red lines indicate the time point of recurrence in rUTI patients. (d) Box plot of the numbers of uropathogen CFU determined by semiquantitative culturing (*x* axis) against the relative abundance of the corresponding species-level taxon (E. coli or P. mirabilis) identified by 16S rRNA sequencing (*y* axis) in all urine samples. The uropathogen identified to be associated with the UTI in a specific sample from each patient is indicated by filled circles (dark green, E. coli; light green, P. mirabilis). Download FIG S4, TIF file, 1.4 MB. Copyright © 2019 Thänert et al. 2019 Thänert et al. This content is distributed under the terms of the Creative Commons Attribution 4.0 International license.

10.1128/mBio.01977-19.10 DATA SET S1 Source data for figures and analysis. Download Data Set S1, XLSX file, 0.2 MB. Copyright © 2019 Thänert et al. 2019 Thänert et al. This content is distributed under the terms of the Creative Commons Attribution 4.0 International license.

### Uropathogens show high levels of resistance to a spectrum of antibiotics used to treat UTIs.

**FIG 3:**
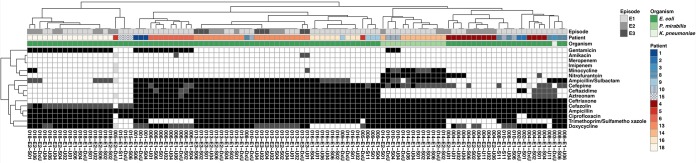
Isolate antibiotic susceptibility profile clusters by patient. A heat map of the results of antibiotic susceptibility testing of all isolates is shown. Profiles of susceptibility to specific antibiotics and individual isolates are hierarchically clustered on the *y* axis and the *x* axis, respectively. Black squares, resistance; dark gray squares, intermediate phenotype; white squares, susceptibility; light gray squares, missing data. The patient and episode of isolation, and the isolate species identity are given as colored annotations on top of the heat map.

We observed high levels of phenotypic resistance against antibiotics commonly used to treat UTIs in all isolates longitudinally collected from the intestinal and urinary tracts (Fig. 3; Data Set S1). Isolates were frequently resistant to ciprofloxacin (97.2%), doxycycline (54.6%), gentamicin (29.6%), trimethoprim-sulfamethoxazole (70.4%), aztreonam (48.1%), cephalosporins (ceftriaxone, 80.6%; cefazolin, 83.3%; ceftazidime, 42.6%; cefepime, 45.4%), and ampicillin-sulbactam (50%). However, we observed no resistance against meropenem, and non-P. mirabilis isolates were only sparsely resistant to nitrofurantoin (5.3%). We did observe a high similarity of the susceptibility profiles of E. coli and P. mirabilis strains cultured from different body habitats or at different time points from the same patient (Fig. 3). Most notably, all 27 isolates cultured from stool and urine specimens from patient 13 during and after three subsequent episodes of symptomatic UTI had similar resistance patterns. The susceptibility profiles of the E. coli isolates from patients 4, 8, and 9 were markedly more variable. This variability was not correlated with the isolate source, indicating that specific within-clone genetic differences might contribute to the observed difference in their susceptibility profiles.

**FIG 4:**
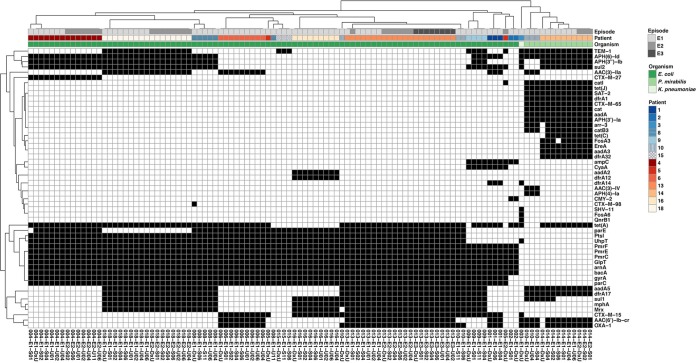
Isolate antibiotic gene repertoire clusters by patient. A heat map of all resistance genes identified by use of the RGI software and the ResFinder database in the genomes of all isolates is shown. Resistance genes and individual isolates are hierarchically clustered on the *y* axis and *x* axis, respectively. Black squares, the presence of a specific resistance gene. The patient and episode of isolation, and the isolate species identity are given as colored annotations on top of the heat map.

Querying the isolate whole-genome sequences for acquired antibiotic resistance genes (ARGs) using the RGI software and the ResFinder database, we identified key differences for the isolates cultured from patients 8 and 9 that explained most of the observed phenotypic variation (Fig. 3 and 4; Data Set S1). We identified the absence of *tetA*, mediating resistance against tetracyclines, as a potential reason for the increased susceptibility of the DxU isolate of patient 9 (9-E1-DxU) to doxycycline. Also, we identified the *bla*_CTX-M-98_ β-lactamase solely in the DxU isolate of patient 8 (8-E1-DxU) that was resistant to all tested cephalosporins, while isolates from all asymptomatic time points harbored *bla*_CTX-M-27_ and were susceptible to cefepime and ceftazidime. These observed differences, as well as the observation that the genomes of individual isolates (isolates 8-E1-U11 and 9-E1-DxU) encoded fewer ARGs than all other isolates from the same patient, indicate that the absence of specific mobile genetic elements or plasmids might cause an altered resistance profile of individual isolates.

As expected, all but 22 isolates (87/109, 79.8%) harbored ESBL-encoding genes (Fig. 4). Of these, the globally most widely distributed ESBL, *bla*_CTX-M-15_ (50/109), was most frequently identified. Additionally, β-lactamase genes *bla*_OXA-1_ (48/109) and *bla*_TEM-1_ (39/109) were frequently identified in the isolates, and *bla*_OXA-1_ was commonly present in isolate genomes with *bla*_CTX-M-15_ (36/50). These isolates exhibited increased resistance against third- and fourth-generation cephalosporins, like cefepime and ceftazidime, highlighting the rise of resistance against later-generation β-lactams (Fig. 3). Moreover, emerging mutations in the *parC* and the *gyrA* genes, associated with broad-spectrum resistance against fluoroquinolones, were virtually omnipresent in E. coli isolates (93/95). Overall, the P. mirabilis isolates harbored the most diverse resistance gene repertoire, including as many as 26 unique ARGs, which frequently included genes for multiple ESBLs.

### Within-patient uropathogen populations share resistance plasmids and virulence genes.

Integrating three tools used to identify plasmidic elements (the PlasmidFinder, Recycler, and PlasmidSpades software) with *in silico* ARG typing (see Materials and Methods), we observed that the fraction of mobilized ARGs was similar for the majority of E. coli isolates cultured from the same patient irrespective of their source (Fig. S5). While point mutations that mediate resistance against fluoroquinolones, fosfomycin, and peptide antibiotics were exclusively typed to chromosomal locations, the majority of ARGs were identified to be located on plasmidic elements (Fig. S5). Genes for resistance to aminoglycosides, macrolides, sulfonamides, and trimethoprim were exclusively identified on putative plasmid contigs (Fig. S5). To determine whether the within-patient AR E. coli community shared a pool of common resistance plasmids, we determined average nucleotide identity and coverage for each pairwise alignment of all identified resistance plasmids from all isolates (see Materials and Methods and Data Set S1). Hierarchical clustering of pairwise similarity indicated highly similar resistance plasmid pools for isolates cultured from the same patient (Fig. S6), with the exception of single isolates collected from patients 8 and 9. *In silico* ARG typing showed that these plasmidic elements harbored the same resistance genes (Fig. S6). These observations indicate that a defined pool of resistance plasmids shared by the majority of AR E. coli clones colonizing the intestinal and urinary tracts of an individual determines their susceptibility profile, which is similarly shared population wide.

10.1128/mBio.01977-19.5 FIG S5 Similar proportions of ARGs are typed to chromosomal and plasmidic locations for the majority of within-patient isolates. ARGs with a putative plasmid location were identified by running the RGI software and using the ResFinder database on all putative plasmid contigs. All ARGs not identified in that pool were assigned to a chromosomal location. Download FIG S5, TIF file, 2.0 MB. Copyright © 2019 Thänert et al. 2019 Thänert et al. This content is distributed under the terms of the Creative Commons Attribution 4.0 International license.

10.1128/mBio.01977-19.6 FIG S6 Antimicrobial-resistant E. coli isolates colonizing the intestinal and urinary tracts share a pool of resistance plasmids. (Top) Hierarchical clustering of the putative resistance plasmids of all isolates based on the Hadamard matrix, comprising the average nucleotide identify and coverage. The source of each isolate (patient and specimen type) is given as a colored annotation at the bottom of the dendrogram. (Bottom) Putative resistance genes identified by the RGI software and using the ResFinder database on all putative resistance plasmids are depicted. Resistance genes are grouped by antibiotic class on the *y* axis. Black squares indicate the presence of a specific resistance gene in the resistance plasmid pool of an isolate. Download FIG S6, TIF file, 0.7 MB. Copyright © 2019 Thänert et al. 2019 Thänert et al. This content is distributed under the terms of the Creative Commons Attribution 4.0 International license.

Hierarchical clustering based on average nucleotide identity (Fig. S6) indicated that one isolate cultured from each of patients 8 (isolate 8-E1-U11) and 9 (isolate 9-E1-DxU) exhibited a significant change in the pool of resistance plasmids from that in all other isolates collected from the same patient. Notably, the pool of ARGs typed to putative plasmidic elements varied substantially for these isolates, with isolate 9-E1-DxU missing the genes *aph(3ʺ)-Ib* and *aph(*6*)-Id* and isolate 8-E1-E11 solely retaining *bla*_CTX-M-27_ (Fig. S6). These observations indicate that plasmid replacement occurred in the uropathogenic E. coli lineages cultured from patients 8 and 9, resulting in altered clinical resistance profiles (Fig. 3; Fig. S6).

*In silico* characterization of the virulence profile of E. coli isolates showed that isolates cultured from the same patients clustered together, indicating that all isolates from the same lineage share the same putative urovirulence factors (PUFs) (Fig. S7; Data Set S1). Genes encoding key virulence factors, like the fimbrial adhesin FimH (100% of isolates), iron acquisition factors (Iha, 97.8%; FyuA, 100%; Irp2, 100%; ChuA, 100%; FeoB, 100%), or the bacteriocin Usp (93.7%), were virtually omnipresent among the drug-resistant E. coli isolates. No virulence factors were restricted to or significantly associated with isolates cultured from rUTI patients. Similarly, isolates cultured from rUTI patients did not harbor more total virulence factors than non-rUTI isolates, indicating that no set of specific virulence factors was associated with rUTIs.

10.1128/mBio.01977-19.7 FIG S7 Isolates cultured from the same patient have similar putative urovirulence factor gene repertoires. PUF genes and individual isolates are hierarchically clustered on the *y* axis and *x* axis, respectively. Black squares indicate the presence of a specific PUF gene. The episode and patient of isolation, as well as the isolate phylogroup group, are given as colored annotations on top of the heat map. Download FIG S7, TIF file, 0.5 MB. Copyright © 2019 Thänert et al. 2019 Thänert et al. This content is distributed under the terms of the Creative Commons Attribution 4.0 International license.

## DISCUSSION

The rise of antibiotic resistance in uropathogens has complicated the effective treatment of UTIs (6, 10). The rate-limiting side effects of antimicrobials as well as limited strategies to identify patients at risk of a recurrence increase the mortality and morbidity associated with UTIs caused by AROs (26). Previous studies have identified the intestinal tract as a reservoir for uropathogens (11, 27), highlighting the relevance of intestinal AR pathogen colonization for the pathology of UTIs. However, there is a lack of prospective studies determining the transmission dynamics of drug-resistant uropathogens between their intestinal reservoir and the urinary tract within individual patients. Importantly, AR uropathogens might have unique patterns of recurrence compared to their susceptible counterparts, as AR organisms have been shown to be enriched following antibiotic interventions that cause the disruption of nonresistant members of the microbiome. Studies focused on AR uropathogens are essential, as they (i) have the potential of informing therapy that aims to limit the selection for drug resistance by inappropriate treatment of AR UTIs, (ii) can identify patients at risk of a recurrence, and (iii) guide the development of novel approaches preventing chronic, difficult-to-treat UTIs. Here, we combined temporally resolved semiquantitative culture of stool and urine samples with genomic interrogation of the corresponding drug-resistant isolates to characterize the patterns of uropathogen persistence and characterize the uropathogen populations in rUTI and non-rUTI patients genotypically and phenotypically.

In this study, we predominantly recovered isolates of the same sequence type (ST) from stool and urine samples from each patient (11/12 patients). In a single patient, patient 5, we observed that consecutive episodes of UTI were associated with E. coli isolates from different phylogroups (ST131 phylogroup B2, ST69 phylogroup D). This trend observed for within-patient sequence type homogeneity contradicts the findings of previous studies that have shown substantial within-patient sequence type variability of fecal ESBL-producing E. coli colonization or UPEC populations (11, 28, 29). As these studies mostly utilized sampling schemes similar to the one applied in this study (11, 29), our observation of a single dominant sequence type per patient might be unique for the pathophysiology of UTIs caused by AROs. The dominance of ST131, which is frequently identified to be the predominant sequence type in cross-sectional studies of drug-resistant infections (20) and was identified for 76.5% (13/17) of isolates collected during symptomatic infections in this study, as well as the lack of a dominant sequence type associated with more susceptible isolates causing UTIs, might explain why our observations deviate from the results of prior studies. While the structure and dynamics of intestinal uropathogen populations have been extensively studied (15, 27, 30), less is known about the population structure of intestinal AROs with uropathogenic potential. Characterization of pairwise SNP distances between isolates collected during UTI as well as at asymptomatic time points revealed interpatient differences in the clonal diversity of the AR *Enterobacteriaceae* populations in the intestine and the urinary tract (Fig. 1B; see also Fig. S1 in the supplemental material). Thus, while the uropathogen populations of most patients (10/14, patients 1, 2, 4, 6, 8, 13, 14, 15, 16, and 18) comprised isolates of a single lineage separated by an average SNP distance of <15, the E. coli populations of patients 9 and 10 were markedly more diverse (average SNP distance, >50). Interpatient differences in the clonal diversity of intestinal ARO populations have previously been reported (10, 29) and could indicate that while a single acquisition event may result in intestinal ARO colonization in the majority of patients, individual patients may be predisposed to *de novo* acquisitions of resistant organisms or colonized by hypermutating strains. While a change in the rate of transition/transversion, often associated with hypermutating lineages of E. coli (31), was not observed for the isolates collected from patients 9 and 10, the predominance of base transitions over transversions observed here is consistent with the patterns observed in hypermutating lineages with a defective methyl-directed mismatch repair system (32). Moreover, UTIs are known to be associated with higher frequencies of hypermutators (33), making this the most likely explanation for the higher SNP distance observed for patients 9 and 10. Similar to previous results (34), we observed that the degree of isolate core genome relatedness was explained by the host and not by the habitat, indicating the lack of substantial genomic signatures associated with adaptation specifically to either the intestinal tract or the urinary tract. This is further supported by previous studies highlighting that some of the same virulence factors that mediate urinary tract colonization also increase the intestinal persistence of uropathogens (35, 36). Moreover, we found that specific clones of E. coli and P. mirabilis were isolated from urine as well as stool samples, highlighting that these uropathogens are well adapted to both habitats and suggesting continuous or intermittent transmission between these ARO niches.

Historically, identification and genomic comparison of strains associated with rUTIs to previous isolates from the same patient have been used to gain insights into the mode of recurrence (37). A multitude of studies have reported variable proportions of rUTIs that are associated with the clone from the first episode (same-strain rUTIs) and different-strain rUTIs (10, 11, 27, 37). Our approach of combining serial semiquantitative stool and urine culture with genomic characterization of the associated isolates allowed us to overcome the limitations associated with the lack of longitudinal sample collection and gain unique, temporally resolved insights into the patterns of uropathogen persistence following UTIs. We observed only a single case of a different-strain rUTI (patient 5; Fig. 1). Interestingly, all stool samples cultured from this patient were negative for AR *Enterobacteriaceae*, indicating that introduction from an extraintestinal source might have caused the recurrence in this patient.

Several reports in mice and humans have implicated persistent bacteriuria as an outcome of UTIs (1, 38–40). However, it remains controversial whether asymptomatic bacteriuria predisposes patients to rUTIs, and different reports have either supported (14) or refuted (15, 41) this assumption. Here, we observed persistently high urine titers of AR E. coli (>1 × 10^5^ CFU/ml) in two patients (patients 4 and 6) that subsequently experienced an episode of rUTI within 180 days after the conclusion of antimicrobial therapy. Interestingly, the intestinal and urinary tracts of both patients were colonized by a single lineage of E. coli. As the SNP distance between the urine isolates was low (<15 SNPs) and no other lineage of AR E. coli was isolated at any time point preceding the recurrence, our observation implicates asymptomatic bacteriuria in the persistence of AR uropathogens following UTIs. 16S rRNA sequencing confirmed the persistent uropathogen dominance in the urinary microbiomes of these two patients. Recently, it has been proposed that asymptomatic bacteriuria might represent a state of dysbiosis on a spectrum of urinary microbiome health that prevents the restoration of bladder homeostasis (42). This new paradigm and studies of host-pathogen interactions following infectious episodes (43) might help provide an understanding of how infections can be followed by a phase of resilience to symptomatic disease, even though the uropathogen is never cleared from the urinary tract and might cause subsequent episodes of rUTIs.

With the observation that isolates genetically identical to UTI-associated E. coli isolates could be found in the intestine (11, 27), periurethral contamination prior to urethral introduction has become generally accepted as a mechanism of uropathogen entry prior to UTIs. Here, we show that the uropathogen populations in the intestinal reservoir and urinary tract are highly interconnected. In four patients (patients 6, 13, 14, and 18) afflicted by rUTIs and one patient that experienced only a single episode of UTI (patient 8), we isolated the same clone (SNP distance = 0) from urine as well as from stool samples (Fig. S1). Moreover, in four patients that later experienced rUTIs (patient 13, 14, 16, and 18), recurrences were preceded by at least one sterile urine sample. However, we still isolated from fecal samples AR uropathogens at the time of culture-negative urine collection, and the lineage associated with the recurrence was isolated from stool samples prior to the recurrence. This supports the hypothesis that the intestine is a reservoir for uropathogen persistence during antimicrobial therapy (11, 27). Moreover, we show that AR uropathogens resurface in the urinary tract following or coinciding with the occurrence of increased stool titers (Fig. 2). Strikingly, we isolated the same lineage that was associated with recurrences during these intestinal blooms (Fig. 2; Fig. S1 and S3), suggesting that a highly dynamic process of urine clearance, intestinal persistence, and urinary tract reinoculation fuels a cycle of rUTI in some patients. The detection of a similar bloom in a non-rUTI patient (patient 8), however, suggests that intestinal ARO blooms are not invariably succeeded by recurrences. Consistently, prior studies have implied that the interplay of uropathogen and host factors might result in a patient-specific rUTI pathophysiology (39, 44). The observation of repeated AR uropathogen entry into the urinary tract without subsequent persistent urinary tract colonization (patients 8, 9, 10, and 16; Fig. 2) suggests that the pathophysiology of UTIs involves an iterative process of periurethral contamination, urinary tract colonization, and bacterial clearance (Fig. 2). This observation necessitates an understanding of intestinal AR uropathogen colonization as part of the pathophysiology of rUTIs and advocates for intestinal ARO decolonization as part of routine UTI treatment.

We observed that the majority of cultured AR uropathogens were resistant to trimethoprim-sulfamethoxazole, which are commonly recommended as first-line drugs for UTIs (45). However, we observed resistance against nitrofurantoin only sparsely, indicating that this drug remains an effective first-line option to treat UTIs caused by uropathogenic E. coli strains. The continued efficacy of first-line drugs is critical to maintain the clinical ability to control UTIs caused by AROs, as we commonly observed resistance to important second-line drugs, like ciprofloxacin, levofloxacin, or third- and fourth-generation cephalosporins. Similar trends have been reported in other studies of AR E. coli and P. mirabilis strains worldwide (46–49), highlighting the need for novel treatment options for UTIs in case residual drugs become ineffective. Such novel strategies to treat and prevent UTIs, including vaccination (65), selective depletion of uropathogens from their reservoirs (50), or antivirulence therapies (51), have already been successfully tested in animal models.

While our approach of serially sampling the within-host ARO population in the intestine and the urinary tract allowed us to describe their clonal diversity, the lack of multiple isolates cultured from the same sample limited our ability to describe subspecies ARO population dynamics and probably led to an underestimation of the genetic diversity of the uropathogen populations. Moreover, analysis of multiple clones per sample would enable in-depth analysis of within-host evolution, which has been implicated to be important in the pathophysiology of other infectious diseases. Another limitation of the exploratory study presented here is the small sample size, necessitating validation of our observations in a study with a large cohort of UTI patients. While the findings obtained with the small subset warrant further validation, our observations have a substantial potential for guiding future research avenues. Thus, based on our results, evaluation of the connection of the intestinal microbiome and the pathophysiology of UTIs is a critical next step, as it can provide further insights into the gut-urinary tract axis and help develop novel treatment strategies targeted to deplete the intestinal uropathogen reservoir. In this context, future studies should also evaluate potential differences in the pathophysiology of rUTIs between patients affected by pyelonephritis and patients affected by cystitis, which was beyond the scope of this work. Additionally, the focus of our study on AR uropathogens prevents the direct extrapolation of our findings to the pathophysiology of UTIs caused by susceptible uropathogens, specifically, fluoroquinolone-susceptible organisms. However, as the majority of UTIs are now associated with resistant pathogens (specifically, fluroquinolone-resistant pathogens) in a number of countries (52, 53), our observations are relevant to a substantial subset of UTIs.

Taken together, by combining semiquantitative culturing with the comparative genomics of serially sampled isolates, we provide support for substantial interpatient differences in the pathophysiology of rUTIs caused by AR uropathogens. We demonstrate that reinfection from external sources, urinary persistence, and intestinal persistence are three routes for rUTIs. Moreover, we provide evidence for the repeated transmission of uropathogens between the intestinal reservoir and the urinary tract and evidence that rUTIs are frequently preceded by an intestinal bloom of uropathogens. The data provided in this study expand our understanding of the temporal dynamics of pathogen clearance and persistence following symptomatic infection and highlight the importance of understanding intestinal ARO colonization as part of the pathophysiology of rUTIs. Thus, monitoring of intestinal AR uropathogen colonization could inform the development of novel treatment strategies targeting intestinal decolonization in patients with a history of AR rUTIs.

## MATERIALS AND METHODS

### Study design, study cohort, and sample collection.

This prospective cohort study was conducted to determine the patterns of uropathogen persistence in the intestinal and urinary tracts following UTIs and to determine the genetic diversity, resistance, and virulence profiles of antimicrobial-resistant uropathogen populations in both body habitats. The study was approved by the Washington University Human Research Protection Office. Subjects were recruited from among patients at Barnes-Jewish Hospital (BJH), St. Louis, MO, with positive urine culture results from standard-of-care testing. Patients with a symptomatic UTI and with a urine culture positive for an ESBL-producing or fluoroquinolone-resistant *Enterobacteriaceae* isolate that were diagnosed and treated by a clinician were included in this study. Patients were excluded if they met one of the following conditions: they were <18 years of age; they had >1 organism at a level above the significance threshold detected in urine by the clinical laboratory, recurrent Clostridioides difficile infection, intra-abdominal devices present, neutropenia (absolute neutrophil count, <500 mm^3^), or intestinal mucosal disruption; they were unlikely to survive for 6 months or were pregnant or unwilling/unable to use contraception; they had short gut syndrome; they used intestinal motility medication; they had inflammatory bowel disease, recent abdominal surgery, active typhlitis or diverticulitis, current gastrointestinal graft-versus-host disease, human immunodeficiency virus infection without antiretroviral therapy, or a CD4 count of <200 mm^3^; they were undergoing peritoneal dialysis; or they had cirrhosis with ascites, active intra-abdominal malignancy, percutaneous nephrostomy tubes, a chronic indwelling Foley or suprapubic catheter, chronic ileal conduit, active hepatitis B or C, ureteral stent, or an active kidney stone. Patients with solid organ transplants were included in the study. Subjects submitted stool and urine specimens to the study team at enrollment; the end of UTI antimicrobial treatment; and days 3, 7, 14, and 30 posttreatment. Samples were kept on ice during transport and immediately processed upon arrival to the lab. Written, informed consent was obtained from all patients.

### Culturing protocols.

**(i) Stool culturing.** Stool samples (∼1 g each) collected at enrollment and on days 0, 7, 30, and 180 post-antimicrobial treatment (pAT) were supplemented with an equal amount (wt/vol) of phosphate-buffered saline (PBS) and vortexed to homogenize the samples. Ten 10-fold serial dilutions were prepared in PBS, and 10 μl of each of the first 10 dilutions was streaked onto selective agar (Hardy Diagnostics, Santa Maria, CA, USA) specific to each patient’s identified ARO using a 10-μl calibrated loop. MacConkey (MAC) agar supplemented with ciprofloxacin (10 μg/ml) was used for ciprofloxacin-resistant *Enterobacteriaceae*, while ESBL-producing *Enterobacteriaceae* were cultured on Hardy Diagnostics ESBL agar and MAC agar supplemented with cefotaxime (1 μg/ml). The CFU were enumerated following overnight incubation at 35°C. The identity of the cultured pathogens was confirmed using matrix-assisted laser desorption ionization–time of flight mass spectrometry (MALDI-TOF MS) (Vitek MS; bioMérieux, Durham, NC, USA). Single colonies were diluted in tryptic soy broth (TSB)-glycerol and stored at −80°C for later analysis.

**(ii) Urine culturing.** Available urine specimens collected at diagnosis, enrollment, and days 0, 7, 30, and 180 pAT were directly plated onto MacConkey agar (Hardy Diagnostics, Santa Maria, CA, USA) using a 10-μl calibrated loop to assess bacterial abundance semiquantitatively. The plates were incubated for 20 to 30 h at 35°C. The numbers of CFU were recorded as <10,000 CFU/ml, >10,000 CFU/ml, or >100,000 CFU/ml. The identity of the cultured pathogens was confirmed using MALDI-TOF MS (Vitek MS; bioMérieux, France). Single colonies were diluted in TSB-glycerol and stored at −80°C for later analysis.

### WGS.

For whole-genome sequencing (WGS), a single isolate was chosen from each stool or urine culture positive for each uropathogen. Isolates stored at −80°C were streaked onto a blood agar plate (Hardy Diagnostics, Santa Maria, CA, USA) and incubated at 35°C overnight. The plates were scraped using a sterile cotton swab, and the scraped material was resuspended in deionized water. Total DNA was extracted from isolates using a QIAamp bacteremia DNA kit (Qiagen, Germantown, MD, USA). Genomic DNA (0.5 ng) was used as the input for Illumina sequencing library preparation using a Nextera kit (Illumina, San Diego, CA, USA) (54). The libraries were pooled at an equal concentration and sequenced to a depth of ∼2.5 million reads (2 × 150 bp) on a NextSeq 500 HighOutput platform (Illumina, San Diego, CA, USA). The remaining adapters were removed from demultiplexed reads using the Trimmomatic (v36) trimming tool (55) (parameters, leading, 10; trailing, 10; sliding window, 4:15; minlen, 60), and potential human read contamination was removed using the DeconSeq (v4.3) tool (56) (default parameters). Draft genomes were *de novo* assembled from preprocessed reads using the SPAdes (v3.11.0) algorithm (57) (parameters, -k 21,33,55,77 –careful). The resulting scaffolds.fasta files were used for all downstream analyses. The quality of the assemblies was controlled using the assembly statistics calculated by the use of the tool QUAST (v5.0.2) (58). High-quality assemblies were annotated using Prokka (v1.12) software (default parameters, contigs > 500 bp) to identify open reading frames (59). Publicly available E. coli reference genomes (see Text S1 in the supplemental material) were downloaded from NCBI and annotated as described above. *In silico* multilocus sequence typing (MLST) was performed using the mlst (v2.11) program (60) (default parameters). Sequenced and acquired isolates were analyzed using a variety of computational programs (Text S1).

### Urine DNA extraction and 16S rRNA sequencing and analysis.

Total DNA was extracted from 2 ml of previously frozen urine samples using the QIAamp bacteremia DNA kit (Qiagen, Germantown, MD, USA). Amplicon libraries of the variable region (V4) of the 16S rRNA gene were constructed using the 515F/806R PCR primers, including Illumina adapter sequences, and the Earth Microbiome protocols (61). Twenty-five-microliter reaction mixtures consisting of 10 μl H_2_O, 12.5 μl *Taq* Hot-Start DNA polymerase (TaKaRa, Kusatsu, Japan), 1 μl forward primer (10 μM), 1 μl reverse primer (10 μM), and 0.5 μl template DNA (1 ng/μl) were prepared in 96-well plates. PCRs were performed in triplicate (settings, 98°C for 30 s; 98°C for 10 s, 50°C for 30 s, and 72°C for 30 s for 35 cycles; 72°C for 2 min). A template-less negative-control reaction was carried out for each reaction. PCR product purity was assessed via gel electrophoresis. 16S rRNA gene amplicons were sequenced on an Illumina MiSeq platform (2 × 250 bp). Demultiplexed raw reads were quality filtered and cleaned from chimeric reads, and the feature table was generated using the dada2 denoise-paired option in the Qiime 2 plug-in (62). The feature table was rarefied to a sampling depth of 5,000 reads. Taxonomy was assigned using the SILVA rRNA database (63). To detect microbiota signatures associated with a predisposition for rUTIs, the bacterial compositions in rUTI and non-rUTI patients with insignificant uropathogen colonization were analyzed. For this purpose, an insignificant uropathogen burden was defined as a <0.25% relative uropathogen abundance, as determined by 16S rRNA gene sequencing, and the detection of less than 10,000 CFU by semiquantitative culturing. Additionally, samples collected immediately preceding a recurrence were analyzed to detect the microbiota signatures preceding the recurrence. Therefore, rUTI patients were matched to non-rUTI patients based on the identified uropathogen, and time-matched samples were analyzed. Results were analyzed and visualized in R using the vegan and ggplot2 packages.

### Antibiotic susceptibility testing.

Antimicrobial susceptibility testing was performed on Mueller-Hinton agar (Hardy Diagnostics, Santa Maria, CA, USA) using the Kirby-Bauer disk diffusion method. Antibiotic disks were purchased from Hardy Diagnostics (Santa Maria, CA, USA) and Becton, Dickinson (Franklin Lakes, NJ, USA). Results were interpreted according to CLSI guidelines (64).

### Data availability.

The data sets generated and analyzed during this study are deposited in the NCBI Sequence Read Archive under BioProject accession no. PRJNA530794.
