# Supplementary material for: Comparative Genomics of Antibiotic-Resistant Uropathogens Implicates Three Routes for Recurrence of Urinary Tract Infections
Source: mBio. 2019 Aug 27;10(4):e01977-19. doi: 10.1128/mBio.01977-19 (PMC6712402; doi:10.1128/mBio.01977-19)
Supplement: TEXT S1 [file mBio.01977-19-s0001.docx]

**Core genome analysis**

General feature format files generated by Prokka were used for species specific core-genome alignment with Roary v3.8.0(1) (default parameters, -cd 100), comprising genes shared by all isolates at 95% identity. Two different core-genomes were constructed for *E. coli* isolates. First, all isolates cultured from diagnostic urine samples (DxU isolates) and publicly available reference genomes were used as input for core genome alignment to contextualize the clinical isolates within the broader taxonomy of clinically relevant *E. coli* strains (Reference genomes used: *E. coli* O55:H7 str. CB9615 - GCA_000025165, *E. coli* O55:H7 str. RM12579 - GCA_000245515, *E. coli* O157:H7 str. EC4115 - GCA_000021125, *E. coli* O157:H7 str. TW14359 - GCA_000022225, *E. coli* O157:H7 str. EDL933 - GCA_000006665, *E. coli* Xuzhou21 - GCA_000262125, *E. coli* HS - GCA_000017765, *E. coli* ATCC 8739 - GCA_000019385, *E. coli* B str. REL606 - GCA_000017985, *E. coli* BL21(DE3) - GCA_000009565, *E. coli* UMNK88 - GCA_000212715, *E. coli* ETEC H10407 - GCA_000210475, *E. coli* str. K-12 substr. DH10B - GCA_000019425, *E. coli* BW2952 - GCA_000022345, *E. coli* DH1 - GCA_000023365, *E. coli* str. K-12 substr. W3110 - GCA_000010245, *E. coli* str. K-12 substr. MG1655 - GCA_000005845, *E. coli* APEC O78 - GCA_000332755, *E. coli* O111:H- str. 11128 - GCA_000010765, *E. coli* O26:H11 str. 11368 - GCA_000091005, *E. coli* O103:H2 str. 12009 - GCA_000010745, *E. coli* O139:H28 str. E24377A - GCA_000832005, *E. coli* SE11 - GCA_000010385, *E. coli* W - GCA_000184185, *E. coli* 55989 - GCA_000026245, *E. coli* O104:H4 str. 2011C-3493 - GCA_000299455, *E. coli* Strain: 2009EL-2050 - GCA_003418725, *E. coli* O104:H4 str. 2009EL-2071 - GCA_000299475, *E. coli* SMS-3-5 - GCA_000019645, *E. coli* IAI39 - GCA_000026345, *E. coli* O7:K1 str. CE10 - GCA_000227625, *E. coli* O127:H6 str. E2348/69 - GCA_000026545, *E. coli* SE15 - GCA_000010485, *E. coli* 536 - GCA_000013305, *E. coli* str. APEC 01 - GCA_003028815, *E. coli* S88 - GCA_000026285, *E. coli* IHE3034 - GCA_000025745, *E. coli* UTI89 - GCA_000013265, *E. coli* UM146 - GCA_000148605, *E. coli* ED1a - GCA_000026305, *E. coli* O83:H1 str. NRG 857C - GCA_000183345, *E. coli* LF82 - GCA_000284495, *E. coli* ABU 83972 - GCA_000148365, *E. coli* CFT073 - GCA_003028795, *E. coli* str. 'clone D i2' - GCA_000233875, *E. coli* str. 'clone D i14' - GCA_000233895). Maximum-likelihood phylogenetic trees were constructed from the core-genome alignment using RAxML v8.2.11(2) (parameters: -n raxml_nwk -m GTRGAMMA -f a -N 1000 -x 54321) and visualized using iTOL v4(3). Second, all isolates from clinical samples (urine and stool) were used to construct a second core genome to assess the relatedness of study isolates. The genome of strain UTI89 was included as an outgroup. A maximum-likelihood tree was constructed using RAxML v8.2.11(2) (parameters: -n raxml_nwk -m GTRGAMMA -f a -N 1000 -x 54321) and visualized using iTOL v4(3).

**SNP-calling in isolates collected from the same patient**

To estimate the single nucleotide polymorphism (SNP) distance between isolates collected from the same patient we aligned short reads to a single ‘reference’ assembly per patient and identified SNPs. The SPAdes assembly with the highest quality, as judged by N50-value, the number of contigs and total length of the assembly, was selected as the ‘patient reference’. Shotgun metagenomic reads from all isolates collected from the same patient were aligned to this ‘reference’ using bowtie2 (parameters: -X 2000 --no-mixed --very-sensitive --n-ceil 0,0.01). Candidate SNPs were identified and filtered using BCFtools with the following criteria: (1) minimum read coverage of 10 reads per SNP position, (2) major allele frequency >95% and (3) a FQ-score as determined by BCFtools of -85 or less. SNP distance matrices were generated using custom R and python scripts on vcf files merged per reference. Data was visualized using the R packages ggplot and ape.

***In silico* identification of antimicrobial resistance features and virulence genes**

Antimicrobial resistance genes (ARGs) were annotated *in silico* using RGI v4.0.2(4), retaining ARGs that covered 100% of the reference sequence at >95% sequence homology. Additional resistance genes, not identified by RGI, were annotated using Resfinder v4.0(5) (parameter: -k 95 -l 1 -a aminoglycoside, colistin, fosfomycin, glycopeptide, nitroimidazole, quinolone, rifampicin, tetracycline, beta-lactam, fusidicacid, macrolide, phenicol, sulphonamide, trimethoprim, oxazolidinone). A list of putative urovirulence factors (PUF) of *E. coli* was curated based on previously published literature(6). The genome of all *E. coli* isolates was queried for PUF presence using BLAST against the reference genome of UTI89. PUF were annotated in isolate genomes if a ‘hit’ had >85% nucleotide identity and >90% coverage. Results were visualized using the pheatmap package in R.

***In silico* identification of putative resistance plasmids**

Putative plasmidic elements were identified using a combinatory approach of *in silico*plasmid typing. First, putative plasmids were identified using plasmidSpades v3.11.0(7) (parameters: --plasmid -k 21,33,55,77 –careful) and Recycler v0.6.2(8) (parameters: -k 77 -i True), that work by classifying contigs in assembly graphs by assessing coverage or by incorporating paired-end read mapping and topology to categorize physically separate entities, respectively. Contigs were confirmed to be of plasmidic origin by BLAST against the NCBI plasmid database, and contigs that covered 90% of a plasmid sequence in the database at >90% sequence homology were retained for downstream analysis. Putative plasmid contigs identified by plasmidSpades or Recycler were aligned to the Spades assembly using BLAST. Spades contigs with <50% query coverage by a putative plasmid contig were removed. Secondly, putative plasmid contigs were identified in the SPAdes assembly based on the presence of replicon sequences using plasmidFinder(9) (parameters: -p enterobacteriaceae -k 95.00). Contigs identified this way were merged with the putative plasmid contigs identified by Recycler and plasmidSpades. The merged pool of putative plasmids was validated via BLAST against the NCBI plasmid database, and contigs that covered 90% of a plasmid sequence in the database at >90% sequence homology were retained. RGI v4.0.2(4) and Resfinder v4.0(5) (parameters as described above) were used to identify contigs that harbored resistance genes in the validated pool of putative plasmid contigs, yielding the final pool of putative resistance plasmids used in downstream analysis. All putative resistance plasmids were used to construct a Hadamard matrix, representing the product of the average nucleotide identity and percent genome aligned, using the ANIm method from pyANI(10) (default parameters). Hierarchical clustering in R was used to assess similarity of resistance plasmid pools between isolates.

1. Page AJ, Cummins CA, Hunt M, Wong VK, Reuter S, Holden MTG, Fookes M, Falush D, Keane JA, Parkhill J. 2015. Roary: rapid large-scale prokaryote pan genome analysis. Bioinformatics 31:3691–3693.

2. Stamatakis A. 2014. RAxML version 8: a tool for phylogenetic analysis and post-analysis of large phylogenies. Bioinformatics 30:1312–3.

3. Letunic I, Bork P. 2016. Interactive tree of life (iTOL) v3: an online tool for the display and annotation of phylogenetic and other trees. Nucleic Acids Res 44:W242–W245.

4. Jia B, Raphenya AR, Alcock B, Waglechner N, Guo P, Tsang KK, Lago BA, Dave BM, Pereira S, Sharma AN, Doshi S, Courtot M, Lo R, Williams LE, Frye JG, Elsayegh T, Sardar D, Westman EL, Pawlowski AC, Johnson TA, Brinkman FSL, Wright GD, McArthur AG. 2017. CARD 2017: expansion and model-centric curation of the comprehensive antibiotic resistance database. Nucleic Acids Res 45:D566–D573.

5. Zankari E, Hasman H, Cosentino S, Vestergaard M, Rasmussen S, Lund O, Aarestrup FM, Larsen M V. 2012. Identification of acquired antimicrobial resistance genes. J Antimicrob Chemother 67:2640–2644.

6. Schreiber HL, Conover MS, Chou W-C, Hibbing ME, Manson AL, Dodson KW, Hannan TJ, Roberts PL, Stapleton AE, Hooton TM, Livny J, Earl AM, Hultgren SJ. 2017. Bacterial virulence phenotypes of *Escherichia coli* and host susceptibility determine risk for urinary tract infections. Sci Transl Med 9:eaaf1283.

7. Antipov D, Hartwick N, Shen M, Raiko M, Lapidus A, Pevzner PA. 2016. plasmidSPAdes: assembling plasmids from whole genome sequencing data. Bioinformatics 32:btw493.

8. Rozov R, Brown Kav A, Bogumil D, Shterzer N, Halperin E, Mizrahi I, Shamir R. 2017. Recycler: an algorithm for detecting plasmids from de novo assembly graphs. Bioinformatics 33:475–482.

9. Carattoli A, Zankari E, García-Fernández A, Voldby Larsen M, Lund O, Villa L, Møller Aarestrup F, Hasman H. 2014. In silico detection and typing of plasmids using PlasmidFinder and plasmid multilocus sequence typing. Antimicrob Agents Chemother 58:3895–903.

10. Pritchard L, Glover RH, Humphris S, Elphinstone JG, Toth IK. 2016. Genomics and taxonomy in diagnostics for food security: soft-rotting enterobacterial plant pathogens. Anal Methods 8:12–24.
