# Supplementary figures and images for: Comparative Genomics of Antibiotic-Resistant Uropathogens Implicates Three Routes for Recurrence of Urinary Tract Infections
Source: mBio. 2019 Aug 27;10(4):e01977-19. doi: 10.1128/mBio.01977-19 (PMC6712402; doi:10.1128/mBio.01977-19)

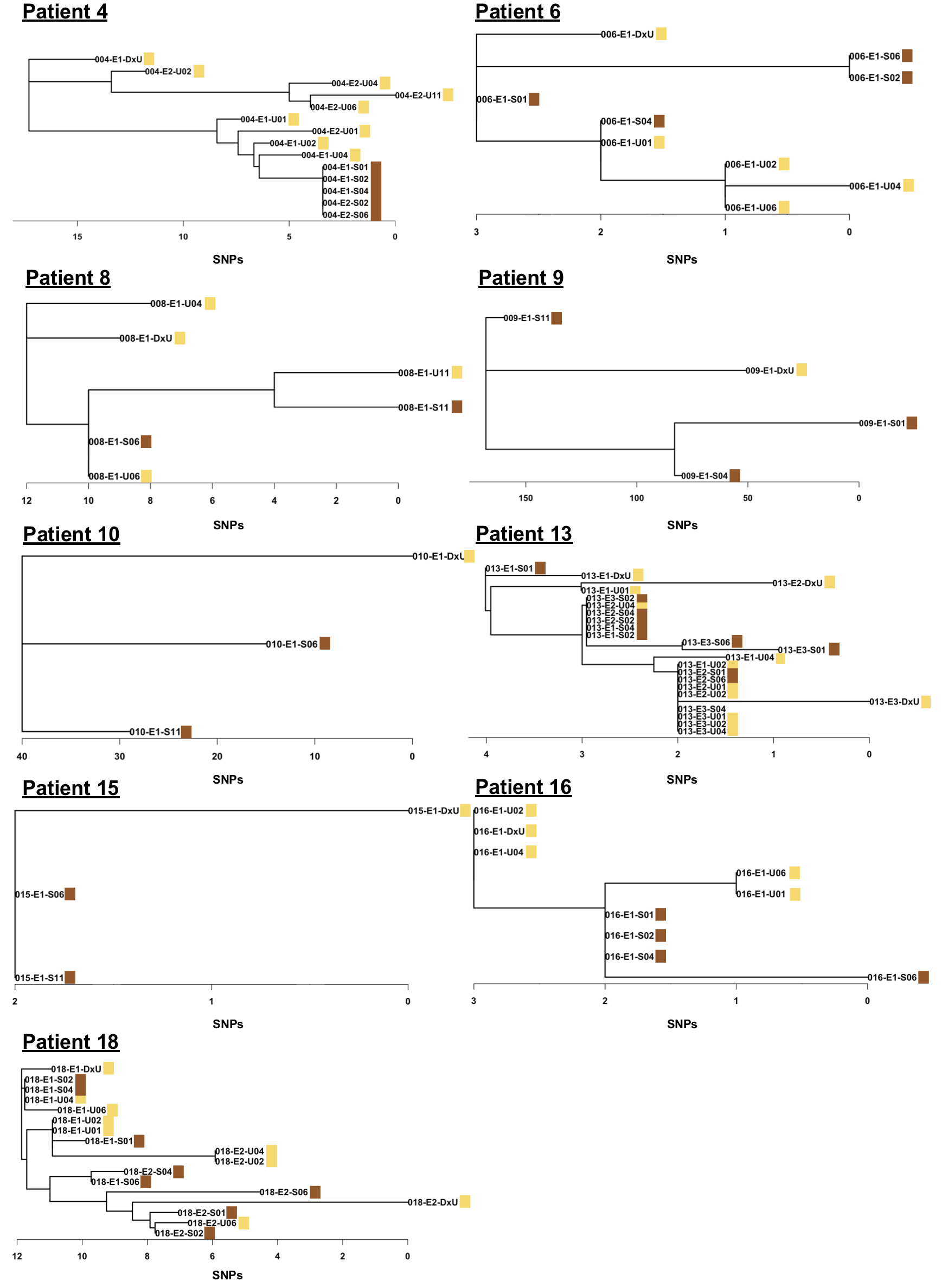

Supplement: FIG S1 [file mBio.01977-19-sf001.tif]

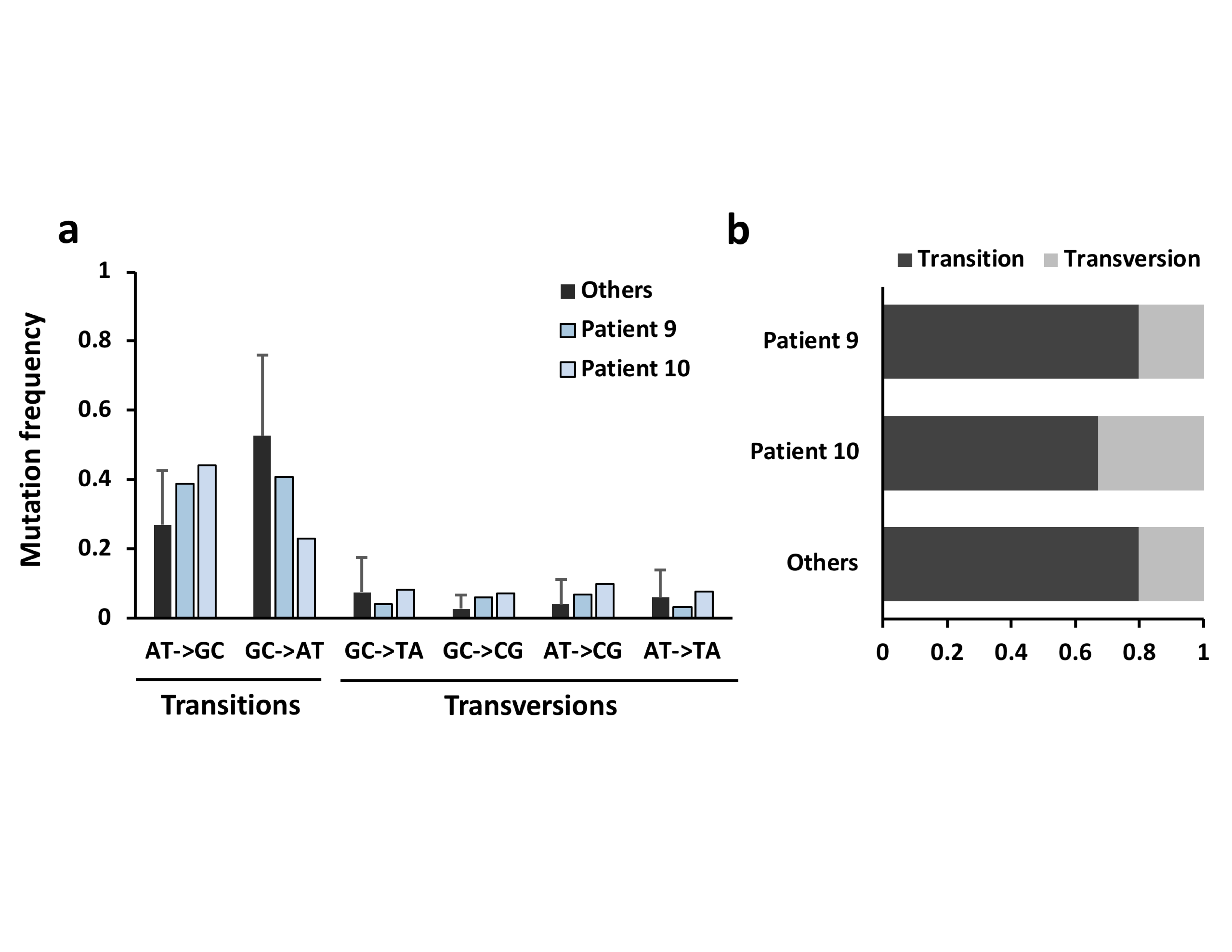

Supplement: FIG S2 [file mBio.01977-19-sf002.tif]

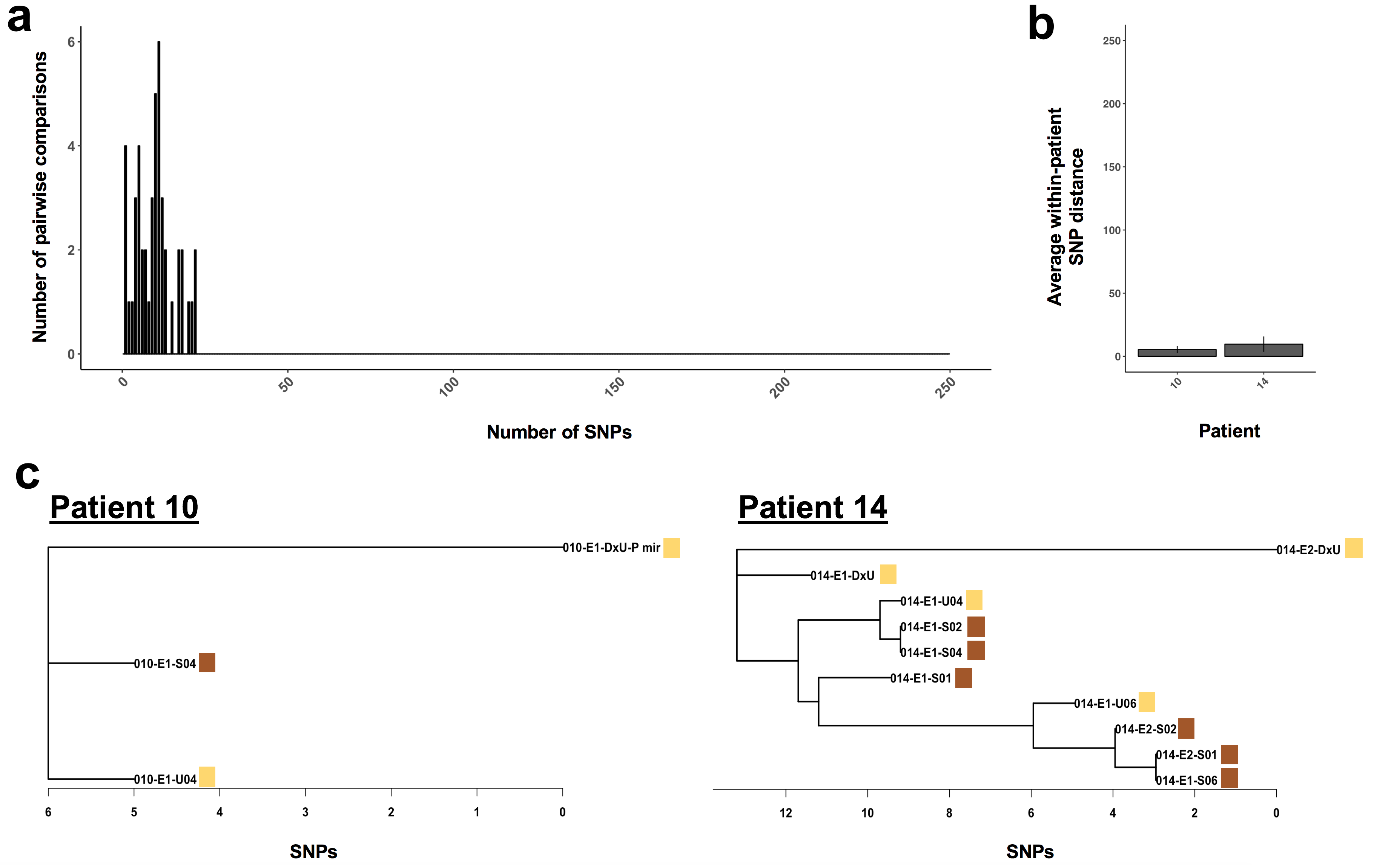

Supplement: FIG S3 [file mBio.01977-19-sf003.tif]

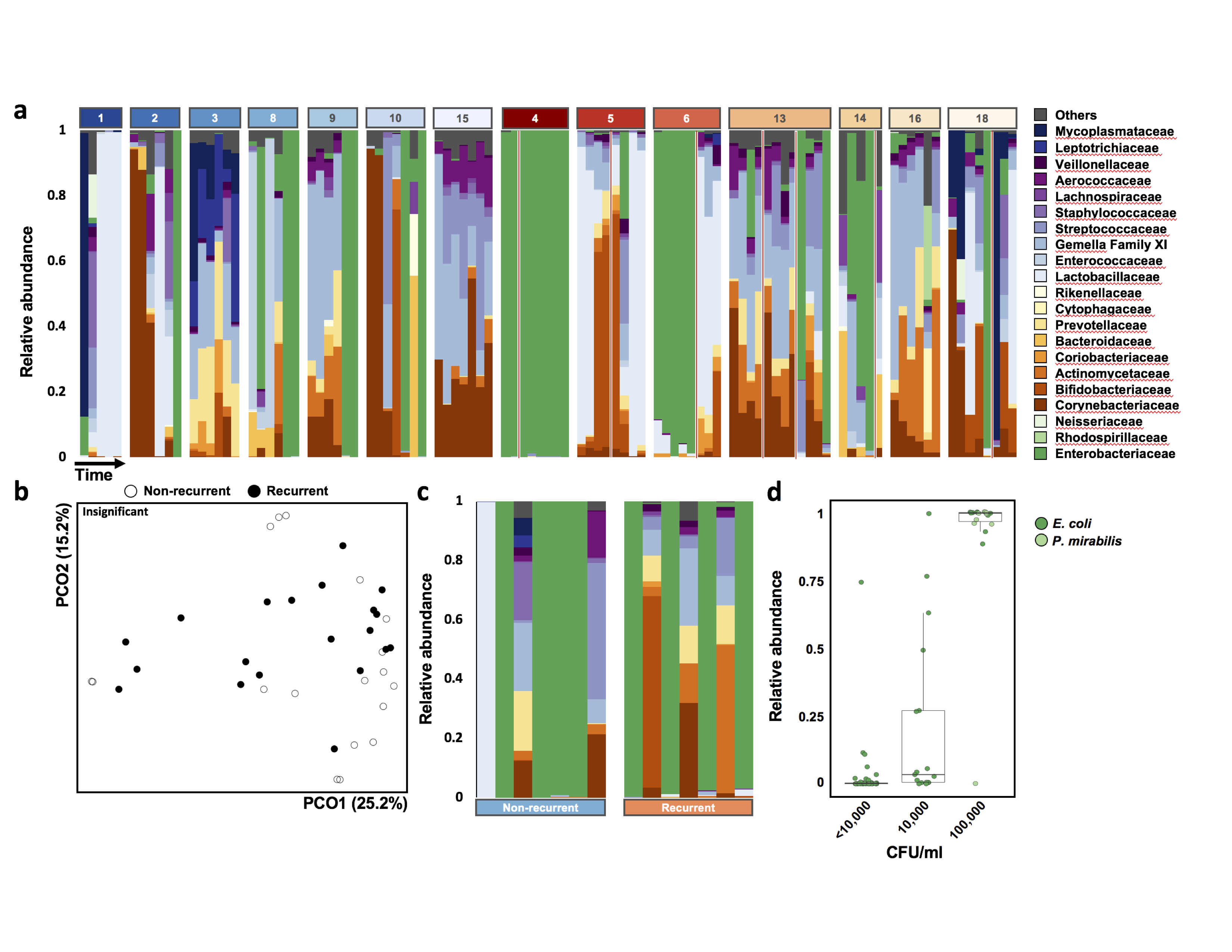

Supplement: FIG S4 [file mBio.01977-19-sf004.tif]

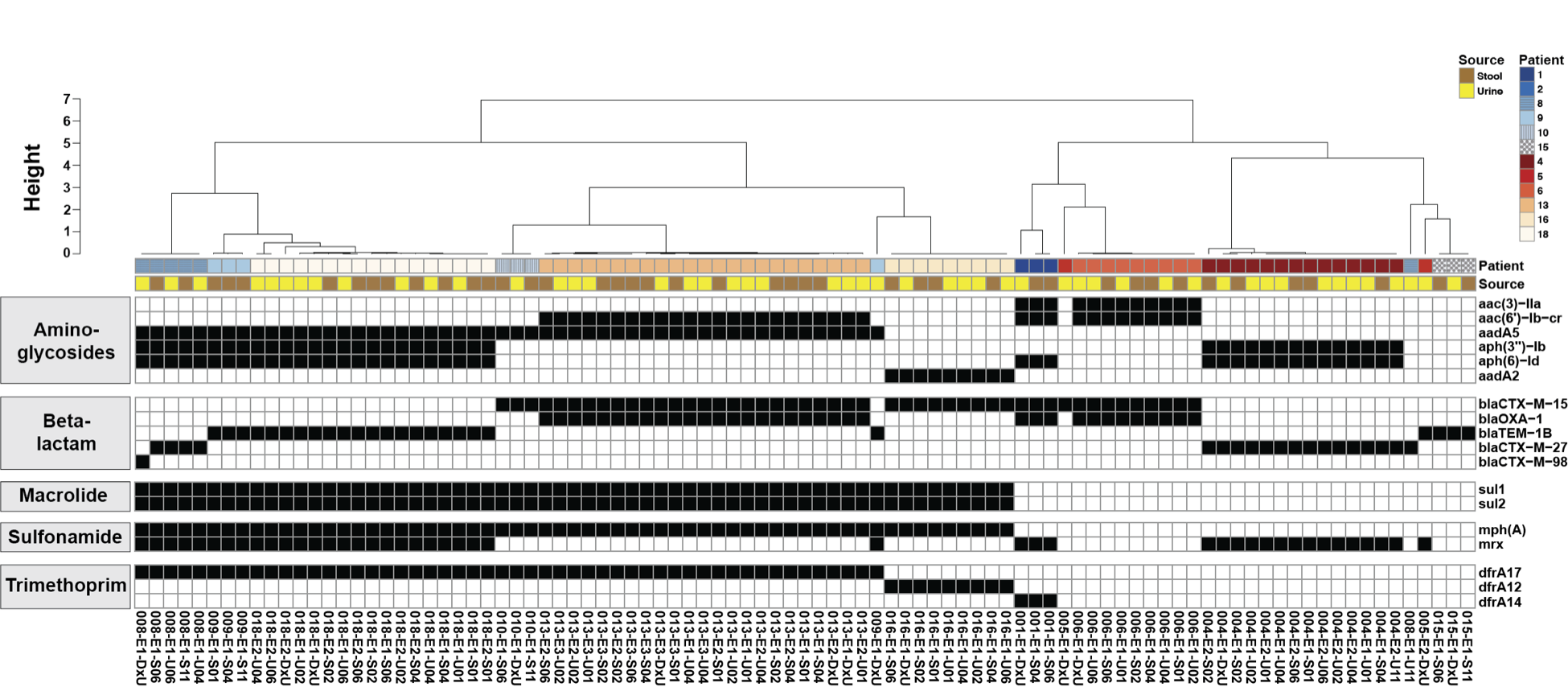

Supplement: FIG S6 [file mBio.01977-19-sf006.tif]

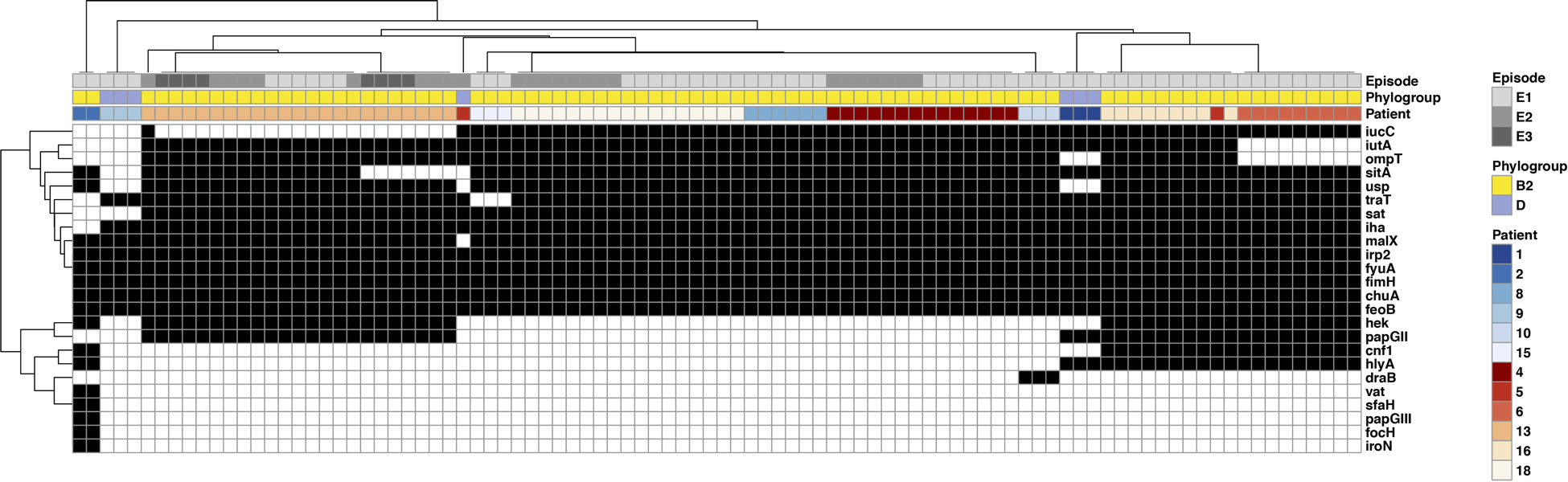

Supplement: FIG S7 [file mBio.01977-19-sf007.tif]
