# Supplementary material for: Comparative Genomics of Antibiotic-Resistant Uropathogens Implicates Three Routes for Recurrence of Urinary Tract Infections
Source: mBio. 2019 Aug 27;10(4):e01977-19. doi: 10.1128/mBio.01977-19 (PMC6712402; doi:10.1128/mBio.01977-19)
Supplement: TABLE S1 [file mBio.01977-19-st001.docx]

| **Patient ID** | **Episode** | **Sample type** | **Timepoint** | **Urine CFU** | **Urine CFU** | **Urine 16s sequenced** | **Isolate ID** |
| --- | --- | --- | --- | --- | --- | --- | --- |
| 1 | 1 | Urine | Diagnostic | 100,000 | - | NA | 001-E1-DxU |
| 1 | 1 | Stool | Enrollment | - | 0 | - | Culture negative |
| 1 | 1 | Stool | 0 days pAT | - | 0 | - | Culture negative |
| 1 | 1 | Stool | 7 days pAT | - | 200 | - | 001-E1-S04 |
| 1 | 1 | Stool | 30 days pAT | - | 500 | - | 001-E1-S06 |
| 1 | 1 | Urine | Enrollment | 0 | - | 001-E1-U01 | Culture negative |
| 1 | 1 | Urine | 0 days pAT | 0 | - | 001-E1-U02 | Culture negative |
| 1 | 1 | Urine | 3 days pAT | NA | - | 001-E1-U03 | NA |
| 1 | 1 | Urine | 7 days pAT | 0 | - | 001-E1-U04 | Culture negative |
| 1 | 1 | Urine | 30 days pAT | 0 | - | 001-E1-U06 | Culture negative |
| 2 | 1 | Urine | Diagnostic | 100,000 | - | NA | 002-E1-DxU |
| 2 | 1 | Stool | Enrollment | - | 10 | - | 002-E1-S01 |
| 2 | 1 | Stool | 0 days pAT | - | 0 | - | Culture negative |
| 2 | 1 | Stool | 7 days pAT | - | 0 | - | Culture negative |
| 2 | 1 | Stool | 30 days pAT | - | 0 | - | Culture negative |
| 2 | 1 | Urine | Enrollment | 0 | - | 002-E1-U01 | Culture negative |
| 2 | 1 | Urine | 0 days pAT | 0 | - | 002-E1-U02 | Culture negative |
| 2 | 1 | Urine | 7 days pAT | 0 | - | 002-E1-U04 | Culture negative |
| 2 | 1 | Urine | 14 days pAT | - | - | 002-E1-U05 | NA |
| 2 | 1 | Urine | 30 days pAT | 0 | - | 002-E1-U06 | Culture negative |
| 2 | 1 | Urine | 60 days pAT | - | - | 002-E1-U07 | NA |
| 3 | 1 | Urine | Diagnostic | 100,000 | - | NA | 003-E1-DxU |
| 3 | 1 | Stool | Enrollment | - | 0 | - | Culture negative |
| 3 | 1 | Stool | 0 days pAT | - | 0 | - | Culture negative |
| 3 | 1 | Stool | 7 days pAT | - | 0 | - | Culture negative |
| 3 | 1 | Stool | 30 days pAT | - | 0 | - | Culture negative |
| 3 | 1 | Stool | 180 days pAT | - | 0 | - | Culture negative |
| 3 | 1 | Urine | Enrollment | 0 | - | 003-E1-U01 | Culture negative |
| 3 | 1 | Urine | 0 days pAT | 0 | - | 003-E1-U02 | Culture negative |
| 3 | 1 | Urine | 7 days pAT | 0 | - | 003-E1-U04 | Culture negative |
| 3 | 1 | Urine | 14 days pAT | - | - | 003-E1-U05 | NA |
| 3 | 1 | Urine | 30 days pAT | 0 | - | 003-E1-U06 | Culture negative |
| 3 | 1 | Urine | 180 days pAT | 0 | - | 003-E1-U11 | Culture negative |
| 4 | 1 | Urine | Diagnostic | 100,000 | - | NA | 004-E1-DxU |
| 4 | 1 | Stool | Enrollment | - | 5,000 | - | 004-E1-S01 |
| 4 | 1 | Stool | 0 days pAT | - | 70,000 | - | 004-E1-S02 |
| 4 | 1 | Stool | 7 days pAT | - | 50,000 | - | 004-E1-S04 |
| 4 | 1 | Urine | Enrollment | 100,000 | - | 004-E1-U01 | 004-E1-U01 |
| 4 | 1 | Urine | 0 days pAT | 100,000 | - | 004-E1-U02 | 004-E1-U02 |
| 4 | 1 | Urine | 7 days pAT | 100,000 | - | 004-E1-U04 | 004-E1-U04 |
| 4 | 2 | Stool | Enrollment | - | 3,700 |  | NA |
| 4 | 2 | Stool | 0 days pAT | - | 80,000 | - | 004-E2-S02 |
| 4 | 2 | Stool | 30 days pAT | - | 7,000 | - | 004-E2-S06 |
| 4 | 2 | Urine | Enrollment | 100,000 | - | 004-E2-U01 | 004-E2-U01 |
| 4 | 2 | Urine | 0 days pAT | 100,000 | - | 004-E2-U02 | 004-E2-U02 |
| 4 | 2 | Urine | 7 days pAT | 100,000 | - | 004-E2-U04 | 004-E2-U04 |
| 4 | 2 | Urine | 30 days pAT | 100,000 | - | 004-E2-U06 | 004-E2-U06 |
| 4 | 2 | Urine | 180 days pAT | 100,000 | - | 004-E2-U11 | 004-E2-U11 |
| 5 | 1 | Urine | Diagnostic | 100,000 | - | NA | 005-E1-DxU |
| 5 | 1 | Stool | Enrollment | - | 0 | - | Culture negative |
| 5 | 1 | Stool | 0 days pAT | - | 0 | - | Culture negative |
| 5 | 1 | Stool | 7 days pAT | - | 0 | - | Culture negative |
| 5 | 1 | Stool | 30 days pAT | - | 0 | - | Culture negative |
| 5 | 1 | Urine | Enrollment | 0 | - | 005-E1-U01 | Culture negative |
| 5 | 1 | Urine | 0 days pAT | 0 | - | 005-E1-U02 | Culture negative |
| 5 | 1 | Urine | 7 days pAT | 10,000 | - | 005-E1-U04 | NA |
| 5 | 1 | Urine | 30 days pAT | 0 | - | 005-E1-U06 | Culture negative |
| 5 | 2 | Urine | Diagnostic | 100,000 | - | NA | 005-E2-DxU |
| 5 | 2 | Urine | Enrollment | 0 | - | 005-E2-U01 | Culture negative |
| 5 | 2 | Urine | 0 days pAT | 10,000 | - | 005-E2-U02 | NA |
| 5 | 2 | Urine | 30 days pAT | 0 | - | 005-E2-U06 | Culture negative |
| 5 | 2 | Urine | 180 days pAT | 0 | - | 005-E2-U11 | Culture negative |
| 6 | 1 | Urine | Diagnostic | 100,000 | - | NA | 006-E1-DxU |
| 6 | 1 | Stool | Enrollment | - | 23,000 | - | 006-E1-S01 |
| 6 | 1 | Stool | 0 days pAT | - | 10,000 | - | 006-E1-S02 |
| 6 | 1 | Stool | 7 days pAT | - | 14,000 | - | 006-E1-S04 |
| 6 | 1 | Stool | 30 days pAT | - | 15,000 | - | 006-E1-S06 |
| 6 | 1 | Urine | Enrollment | 100,000 | - | 006-E1-U01 | 006-E1-U01 |
| 6 | 1 | Urine | 0 days pAT | 100,000 | - | 006-E1-U02 | 006-E1-U02 |
| 6 | 1 | Urine | 7 days pAT | 100,000 | - | 006-E1-U04 | 006-E1-U04 |
| 6 | 1 | Urine | 30 days pAT | 100,000 | - | 006-E1-U06 | 006-E1-U06 |
| 6 | 1 | Urine | 60 days pAT | NA | - | 006-E1-U07 | NA |
| 6 | 2 | Stool | Enrollment | - | 0 | - | Culture negative |
| 6 | 2 | Stool | 7 days pAT | - | 0 | - | Culture negative |
| 6 | 2 | Stool | 180 days pAT | - | 0 | - | Culture negative |
| 6 | 2 | Urine | Enrollment | 0 | - | 006-E2-U01 | Culture negative |
| 6 | 2 | Urine | 7 days pAT | 0 | - | 006-E2-U04 | Culture negative |
| 6 | 2 | Urine | 180 days pAT | 0 | - | 006-E2-U11 | Culture negative |
| 8 | 1 | Urine | Diagnostic | 100,000 | - | NA | 008-E1-DxU |
| 8 | 1 | Stool | 0 days pAT | - | 0 | - | Culture negative |
| 8 | 1 | Stool | 30 days pAT | - | 4,100,000 | - | 008-E1-S06 |
| 8 | 1 | Stool | 180 days pAT | - | 500,000 | - | 008-E1-S11 |
| 8 | 1 | Urine | Enrollment | 0 | - | 008-E1-U01 | Culture negative |
| 8 | 1 | Urine | 0 days pAT | 0 | - | 008-E1-U02 | Culture negative |
| 8 | 1 | Urine | 7 days pAT | 100,000 | - | 008-E1-U04 | 008-E1-U04 |
| 8 | 1 | Urine | 14 days pAT | - | - | 008-E1-U05 | NA |
| 8 | 1 | Urine | 30 days pAT | 100,000 | - | 008-E1-U06 | 008-E1-U06 |
| 8 | 1 | Urine | 180 days pAT | 10,000 | - | 008-E1-U11 | 008-E1-U11 |
| 9 | 1 | Urine | Diagnostic | 100,000 | - | NA | 009-E1-DxU |
| 9 | 1 | Stool | Enrollment | - | 200 | - | 009-E1-S01 |
| 9 | 1 | Stool | 7 days pAT | - | 28,000 | - | 009-E1-S04 |
| 9 | 1 | Stool | 180 days pAT | - | 20,000 | - | 009-E1-S11 |
| 9 | 1 | Urine | Enrollment | 0 | - | 009-E1-U01 | Culture negative |
| 9 | 1 | Urine | 0 days pAT | 0 | - | 009-E1-U02 | Culture negative |
| 9 | 1 | Urine | 7 days pAT | 10,000 | - | 009-E1-U04 | NA |
| 9 | 1 | Urine | 14 days pAT | - | - | 009-E1-U05 | NA |
| 9 | 1 | Urine | 30 days pAT | 100,000 | - | 009-E1-U06 | NA |
| 9 | 1 | Urine | 180 days pAT | 100,000 | - | 009-E1-U11 | NA |
| 10 | 1 | Urine | Diagnostic | 100,000 | - | NA | 010-E1-DxU-E_coli, 010-E1-DxU-P_mir |
| 10 | 1 | Stool | Enrollment | - | 0 | - | Culture negative |
| 10 | 1 | Stool | 0 days pAT | - | 0 | - | Culture negative |
| 10 | 1 | Stool | 7 days pAT | - | 30 | - | 010-E1-S04 |
| 10 | 1 | Stool | 30 days pAT | - | 24,000 | - | 010-E1-S06 |
| 10 | 1 | Stool | 180 days pAT | - | 100 | - | 010-E1-S11 |
| 10 | 1 | Urine | Enrollment | 0 | - | 010-E1-U01 | Culture negative |
| 10 | 1 | Urine | 0 days pAT | 0 | - | 010-E1-U02 | Culture negative |
| 10 | 1 | Urine | 7 days pAT | 0 | - | 010-E1-U04 | 010-E1-U04 |
| 10 | 1 | Urine | 14 days pAT | - | - | 010-E1-U05 | NA |
| 10 | 1 | Urine | 30 days pAT | 100,000 | - | 010-E1-U06 | NA |
| 10 | 1 | Urine | 150 days pAT | - | - | 010-E1-U10 | NA |
| 10 | 1 | Urine | 180 days pAT | 100,000 | - | 010-E1-U11 | NA |
| 13 | 1 | Urine | Diagnostic | 100,000 | - | NA | 013-E1-DxU |
| 13 | 1 | Stool | Enrollment | - | 500,000 | - | 013-E1-S01 |
| 13 | 1 | Stool | 0 days pAT | - | 140,000 | - | 013-E1-S02 |
| 13 | 1 | Stool | 7 days pAT | - | 1,100,000 | - | 013-E1-S04 |
| 13 | 1 | Urine | Enrollment | 0 | - | 013-E1-U01 | 013-E1-U01 |
| 13 | 1 | Urine | 0 days pAT | 10,000 | - | 013-E1-U02 | 013-E1-U02 |
| 13 | 1 | Urine | 7 days pAT | 10,000 | - | 013-E1-U04 | 013-E1-U04 |
| 13 | 1 | Urine | 14 days pAT | - | - | 013-E1-U05 | NA |
| 13 | 2 | Urine | Diagnostic | 100,000 | - | NA | 013-E2-DxU |
| 13 | 2 | Stool | Enrollment | - | 500,000 | - | 013-E2-S01 |
| 13 | 2 | Stool | 0 days pAT | - | 5,000,000 | - | 013-E2-S02 |
| 13 | 2 | Stool | 7 days pAT | - | 180,000 | - | 013-E2-S04 |
| 13 | 2 | Stool | 30 days pAT | - | 16,000 | - | 013-E2-S06 |
| 13 | 2 | Urine | Enrollment | 10,000 | - | 013-E2-U01 | 013-E2-U01 |
| 13 | 2 | Urine | 0 days pAT | 0 | - | 013-E2-U02 | 013-E2-U02 |
| 13 | 2 | Urine | 7 days pAT | 10,000 | - | 013-E2-U04 | 013-E2-U04 |
| 13 | 2 | Urine | 30 days pAT | 10,000 | - | 013-E2-U06 | NA |
| 13 | 3 | Urine | Diagnostic | NA | - | NA | 013-E3-DxU |
| 13 | 3 | Stool | Enrollment | - | 2,700,000 | - | 013-E3-S01 |
| 13 | 3 | Stool | 0 days pAT | - | 600,000 | - | 013-E3-S02 |
| 13 | 3 | Stool | 7 days pAT | - | 1,300,000 | - | 013-E3-S04 |
| 13 | 3 | Stool | 30 days pAT | - | 110,000 | - | 013-E3-S06 |
| 13 | 3 | Stool | 180 days pAT | - | 0 | - | Culture negative |
| 13 | 3 | Urine | Enrollment | 10,000 | - | 013-E3-U01 | 013-E3-U01 |
| 13 | 3 | Urine | 0 days pAT | 10,000 | - | 013-E3-U02 | 013-E3-U02 |
| 13 | 3 | Urine | 7 days pAT | 100,000 | - | 013-E3-U04 | 013-E3-U04 |
| 13 | 3 | Urine | 30 days pAT | 100,000 | - | 013-E3-U06 | NA |
| 13 | 3 | Urine | 180 days pAT | 100,000 | - | NA | NA |
| 14 | 1 | Urine | Diagnostic | 100,000 | - | NA | 014-E1-DxU |
| 14 | 1 | Stool | Enrollment | - | 10,000 | - | 014-E1-S01 |
| 14 | 1 | Stool | 0 days pAT | - | 17,000 | - | 014-E1-S02 |
| 14 | 1 | Stool | 7 days pAT | - | 230,000 | - | 014-E1-S04 |
| 14 | 1 | Stool | 30 days pAT | - | 180 | - | 014-E1-S06 |
| 14 | 1 | Urine | Enrollment | 0 | - | 014-E1-U01 | Culture negative |
| 14 | 1 | Urine | 7 days pAT | 0 | - | 014-E1-U04 | 014-E1-U04 |
| 14 | 1 | Urine | 30 days pAT | 10,000 | - | 014-E1-U06 | 014-E1-U06 |
| 14 | 1 | Urine | 150 days pAT | 10,000 | - | 014-E1-U10 | NA |
| 14 | 2 | Urine | Diagnostic | 100,000 | - | NA | 014-E2-DxU |
| 14 | 2 | Stool | Enrollment | - | 10,000 | - | 014-E2-S01 |
| 14 | 2 | Stool | 0 days pAT | - | 27,000 | - | 014-E2-S02 |
| 14 | 2 | Urine | Enrollment | 0 | - |  | Culture negative |
| 14 | 2 | Urine | 0 days pAT | 0 | - | 14-E2-U02 | Culture negative |
| 15 | 1 | Urine | Diagnostic | 100,000 | - | NA | 015-E1-DxU |
| 15 | 1 | Stool | Enrollment | - | 0 | - | Culture negative |
| 15 | 1 | Stool | 0 days pAT | - | 0 | - | Culture negative |
| 15 | 1 | Stool | 7 days pAT | - | 0 | - | Culture negative |
| 15 | 1 | Stool | 30 days pAT | - | 5,000 | - | 015-E1-S06 |
| 15 | 1 | Stool | 180 days pAT | - | 5,000 | - | 015-E1-S11 |
| 15 | 1 | Urine | Enrollment | 0 | - | 015-E1-U01 | Culture negative |
| 15 | 1 | Urine | 0 days pAT | 0 | - | 015-E1-U02 | Culture negative |
| 15 | 1 | Urine | 7 days pAT | 0 | - | 015-E1-U04 | Culture negative |
| 15 | 1 | Urine | 14 days pAT | - | - | 015-E1-U05 | NA |
| 15 | 1 | Urine | 30 days pAT | 0 | - | 015-E1-U06 | Culture negative |
| 15 | 1 | Urine | 90 days pAT | - | - | 015-E1-U08 | NA |
| 15 | 1 | Urine | 180 days pAT | 0 | - | 015-E1-U11 | Culture negative |
| 16 | 1 | Urine | Diagnostic | 100,000 | - | NA | 016-E1-DxU |
| 16 | 1 | Stool | Enrollment | - | 420,000 | - | 016-E1-S01 |
| 16 | 1 | Stool | 0 days pAT | - | 16,000 | - | 016-E1-S02 |
| 16 | 1 | Stool | 7 days pAT | - | 44,000 | - | 016-E1-S04 |
| 16 | 1 | Stool | 30 days pAT | - | 80,000 | - | 016-E1-S06 |
| 16 | 1 | Urine | Enrollment | 0 | - | 016-E1-U01 | 016-E1-U01 |
| 16 | 1 | Urine | 0 days pAT | 10,000 | - | 016-E1-U02 | 016-E1-U02 |
| 16 | 1 | Urine | 3 days pAT | - | - | 016-E1-U03 | NA |
| 16 | 1 | Urine | 7 days pAT | 0 | - | 016-E1-U04 | 016-E1-U04 |
| 16 | 1 | Urine | 30 days pAT | 10,000 | - | 016-E1-U06 | 016-E1-U06 |
| 18 | 1 | Urine | Diagnostic | NA | - | NA | 018-E1-DxU |
| 18 | 1 | Stool | Enrollment | - | 11,000 | - | 018-E1-S01 |
| 18 | 1 | Stool | 0 days pAT | - | 110,000 | - | 018-E1-S02 |
| 18 | 1 | Stool | 7 days pAT | - | 5,000 | - | 018-E1-S04 |
| 18 | 1 | Stool | 30 days pAT | - | 50,000 | - | 018-E1-S06 |
| 18 | 1 | Urine | Enrollment | 0 | - | 018-E1-U01 | 018-E1-U01 |
| 18 | 1 | Urine | 0 days pAT | 100 | - | 018-E1-U02 | 018-E1-U02 |
| 18 | 1 | Urine | 7 days pAT | 10,000 | - | 018-E1-U04 | 018-E1-U04 |
| 18 | 1 | Urine | 30 days pAT | 10,000 | - | 018-E1-U06 | 018-E1-U06 |
| 18 | 1 | Urine | 90 days pAT | - | - | 018-E1-U08 | NA |
| 18 | 2 | Urine | Diagnostic | 100,000 | - | NA | 018-E2-DxU |
| 18 | 2 | Stool | Enrollment | - | 600 | - | 018-E2-S01 |
| 18 | 2 | Stool | 0 days pAT | - | 7,000 | - | 018-E2-S02 |
| 18 | 2 | Stool | 7 days pAT | - | 14,000 | - | 018-E2-S04 |
| 18 | 2 | Stool | 30 days pAT | - | 47,000 | - | 018-E2-S06 |
| 18 | 2 | Urine | Enrollment | 0 | - | 018-E2-U01 | Culture negative |
| 18 | 2 | Urine | 0 days pAT | 10,000 | - | 018-E2-U02 | 018-E2-U02 |
| 18 | 2 | Urine | 7 days pAT | 0 | - | 018-E2-U04 | 018-E2-U04 |
| 18 | 2 | Urine | 30 days pAT | 100,000 | - | NA | 018-E2-U06 |

*NA: Not available

**pAT: post antimicrobial treatment
